# Supplementary material for: Controlled Decoration of [60]Fullerene with Polymannan Analogues and Amino Acid Derivatives through Malondiamide-Based Linkers
Source: Molecules. 2022 Apr 27;27(9):2776. doi: 10.3390/molecules27092776 (PMC9101093; doi:10.3390/molecules27092776)

Supporting Information

Controlled Decoration of [60]Fullerene with Polymannan Analogues and Amino Acid Derivatives through Malondiamide-Based Linkers

Lisa Tanzi, ‡(a,b) Davide Rubes, ‡(a) Teodora Bavaro,(a) Matthieu Sollogoub,(b) Massimo Serra,*(a) Yongmin Zhang,*(b) and Marco Terreni(a)

Table of Contents

S2 Copies of ^{1}H NMR (400 MHz), ^{13}C NMR, HRMS spectra for compound **7**

S4 Copies of ^{1}H NMR (400 MHz), ^{13}C NMR, HRMS spectra for compound **9**

S6 Copies of ^{1}H NMR (400 MHz), ^{13}C NMR, HRMS spectra for compound **10**

S8 Copies of ^{1}H NMR (400 MHz), ^{13}C NMR, HRMS spectra for compound **11**

S10 Copies of ^{1}H NMR (400 MHz), ^{13}C NMR, HRMS spectra for compound **12**

S12 Copies of ^{1}H NMR (400 MHz), ^{13}C NMR, HRMS spectra for compound **13**

S14 Copies of ^{1}H NMR (400 MHz), ^{13}C NMR, HRMS spectra for compound **14**

S16 Copies of ^{1}H NMR (400 MHz), ^{13}C NMR, HRMS spectra for compound **15**

S18 Copies of ^{1}H NMR (400 MHz), ^{13}C NMR, HRMS spectra for compound **16**

S20 Copies of ^{1}H NMR (400 MHz), ^{13}C NMR, HRMS spectra for compound **17**

S22 Copies of ^{1}H NMR (400 MHz), ^{3}C NMR, HRMS spectra for compound **18**

S24 Copies of ^{1}H NMR (400 MHz), ^{13}C NMR, HRMS spectra for compound **19**

S26 Copies of ^{1}H NMR (400 MHz), ^{13}C NMR, HRMS spectra for compound **20**

S28 Copies of ^{1}H NMR (400 MHz), ^{13}C NMR, HRMS spectra for compound **21**

S30 Copies of ^{1}H NMR (400 MHz), ^{13}C NMR, HRMS spectra for compound **22**

S32 Copies of ^{1}H NMR (400 MHz), ^{13}C NMR, HRMS spectra for compound **23**

S34 Copies of ^{1}H NMR (400 MHz), ^{13}C NMR, HRMS, UV-Vis spectra for compound **24**

Compound 7

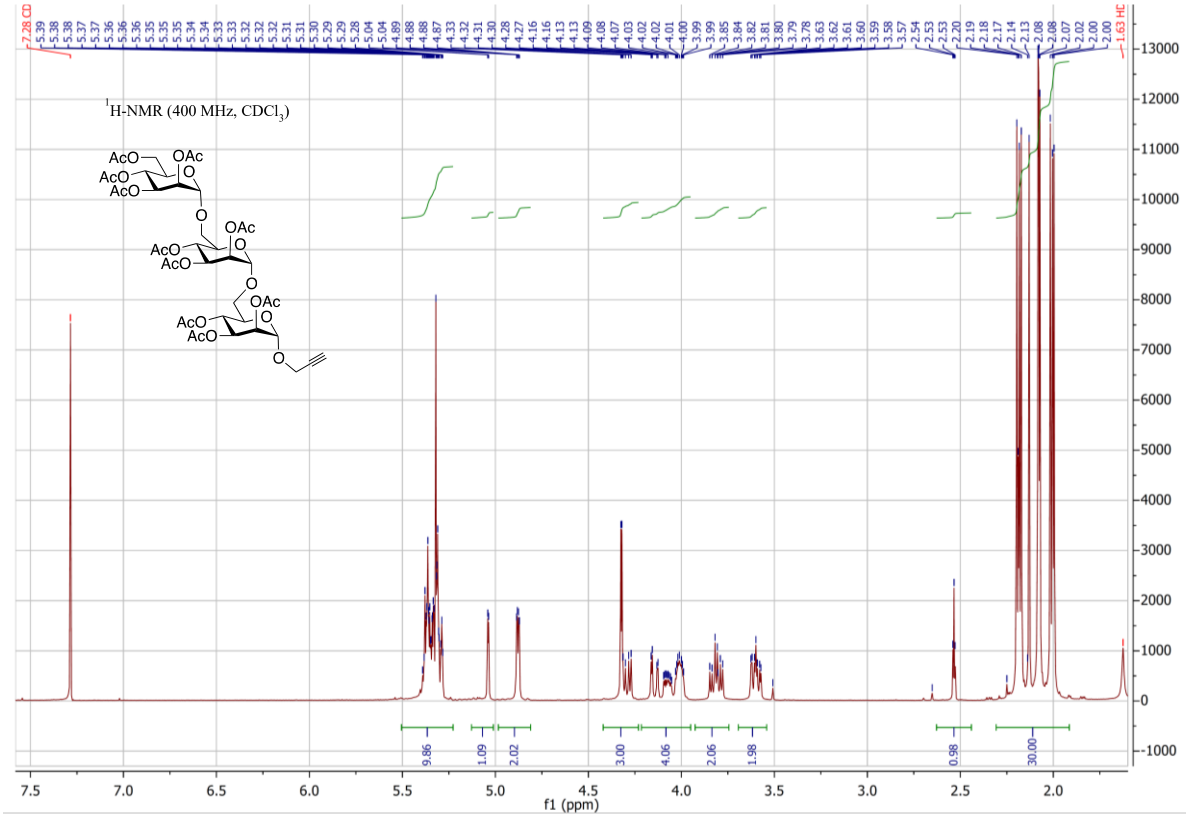

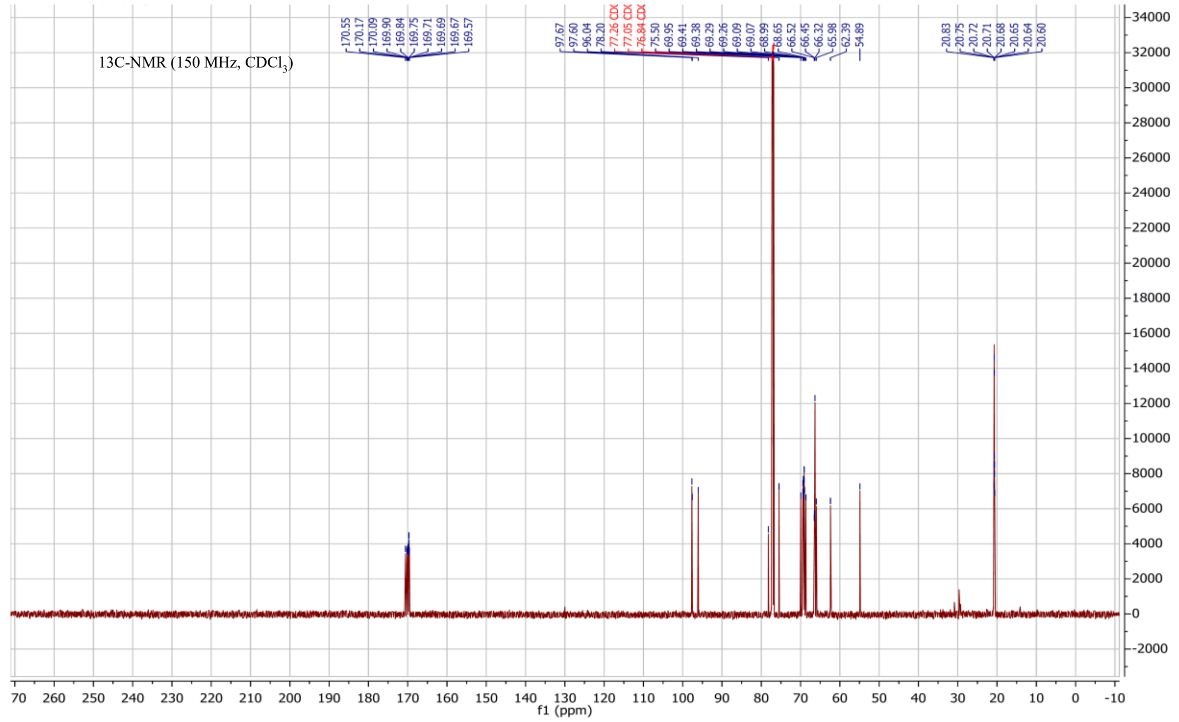

Display Report

Analysis Info

Analysis Name	D:\Data\GOBS\Lisa\LT.tris1,61,6.hs(BIS).d	Acquisition Date	10/7/2019 4:17:59 PM
Method	tune_high.m	Operator	BDAL@DE
Sample Name	LT.tris1,61,6.hs(BIS)	Instrument / Ser#	micrOTOF 235
Comment			

Acquisition Parameter

Source Type	ESI	Ion Polarity	Positive	Set Nebulizer	5.8 psi
Focus	Active			Set Dry Heater	180 °C
Scan Begin	50 m/z	Set Capillary	4500 V	Set Dry Gas	4.0 l/min
Scan End	4000 m/z	Set End Plate Offset	-500 V	Set Divert Valve	Waste

Meas. m/z	#	Formula	m/z	err [ppm]	mSigma	err [mDa]
985.2796	1	C 41 H 54 Na O 26	985.2796	-0.1	22.6	-0.1

Meas. m/z	#	Formula	m/z	err [ppm]	mSigma	err [mDa]

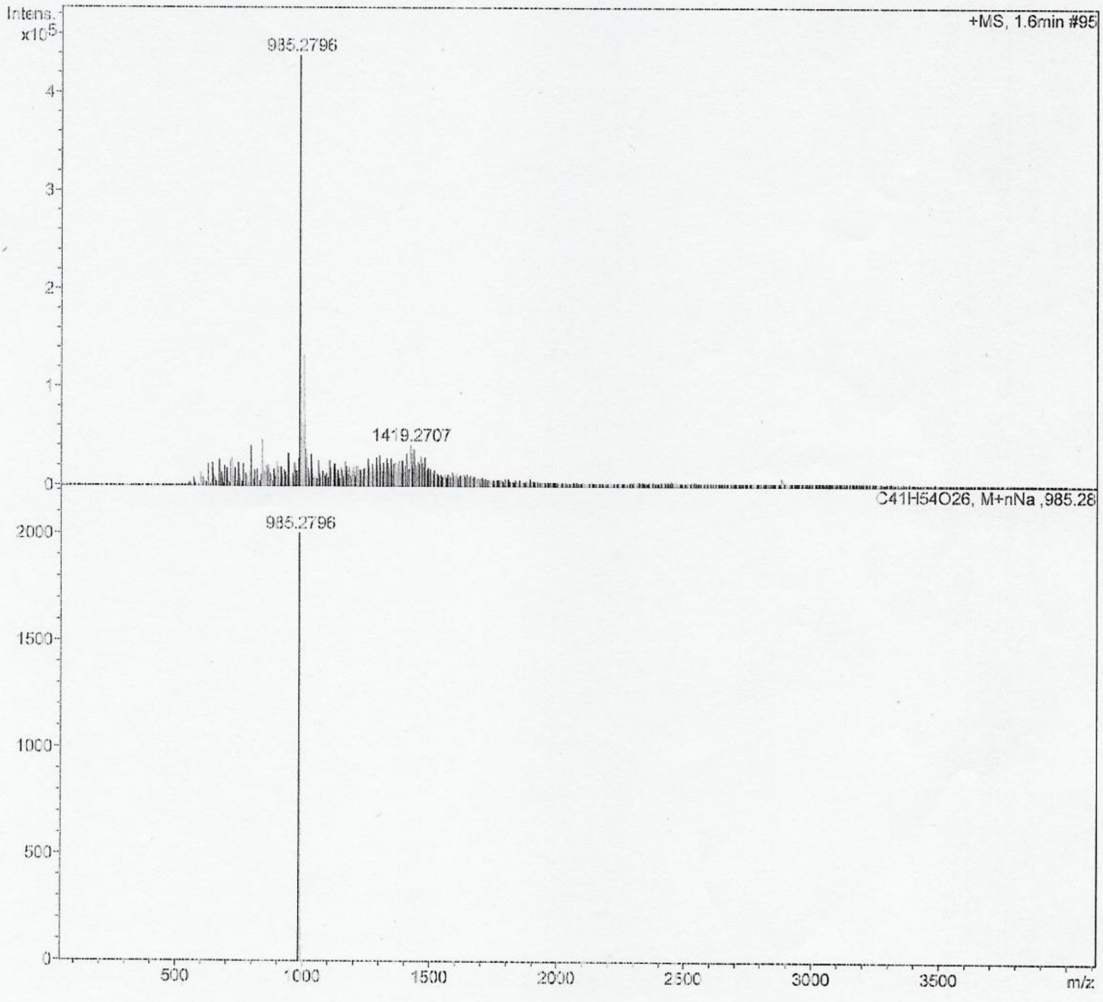

Bruker Compass DataAnalysis 4.0 printed: 10/7/2019 4:37:14 PM Page 1 of 1

Compound 9

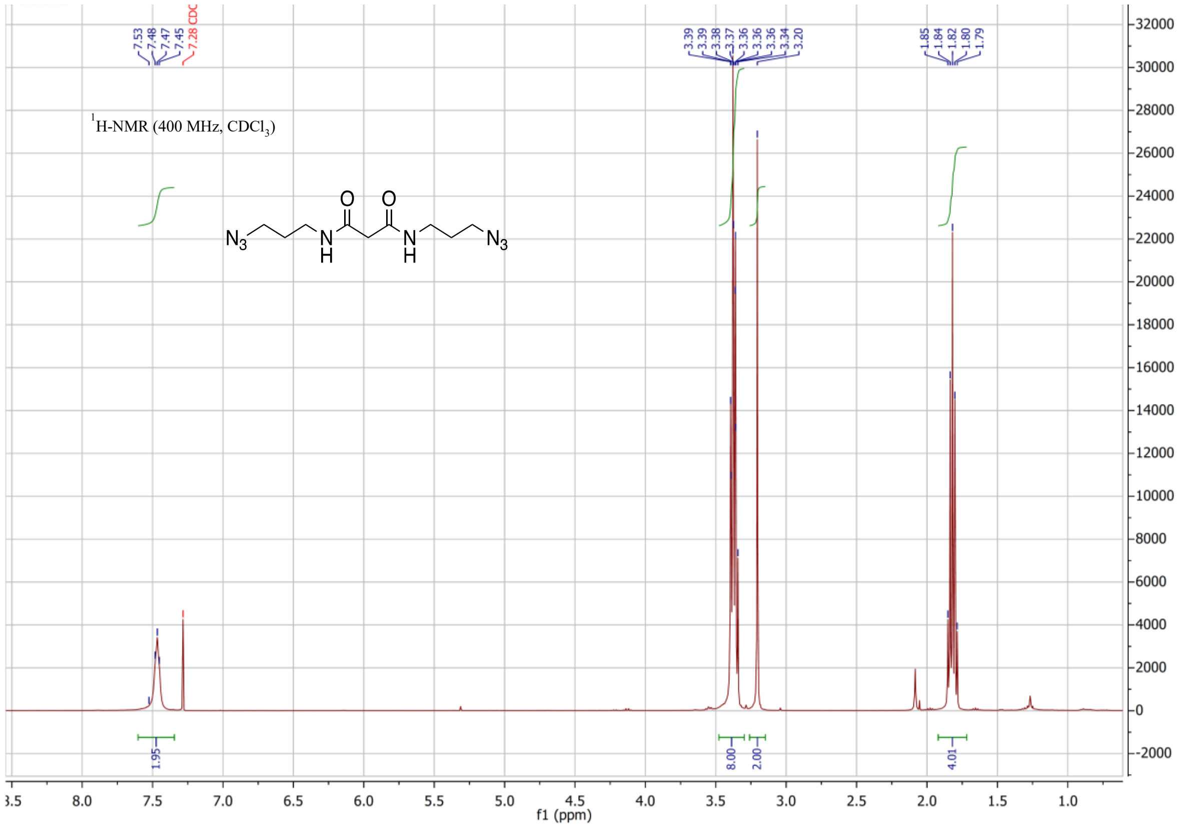
7.53
7.48
7.47
7.45
7.28 CDC
3.39
3.39
3.38
3.37
3.36
3.36
3.36
3.34
3.20
1.85
1.84
1.82
1.80
1.79
1H-NMR (400 MHz, CDCl3)
N3
N
H
O
O
N
H
N3
1.95
8.00
2.00
4.01
f1 (ppm)

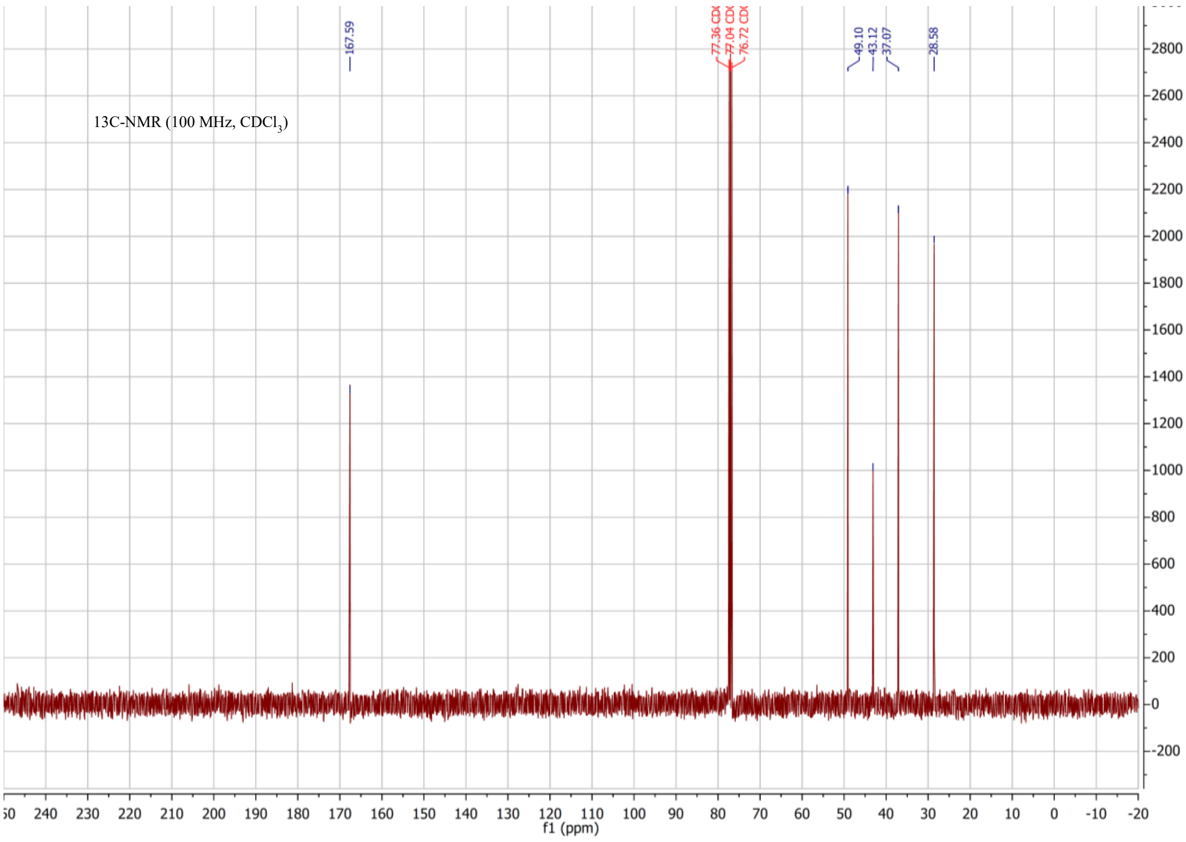
167.59
77.36 CDC
77.04 CDC
76.72 CDC
49.10
43.12
37.07
28.58
13C-NMR (100 MHz, CDCl3)
f1 (ppm)

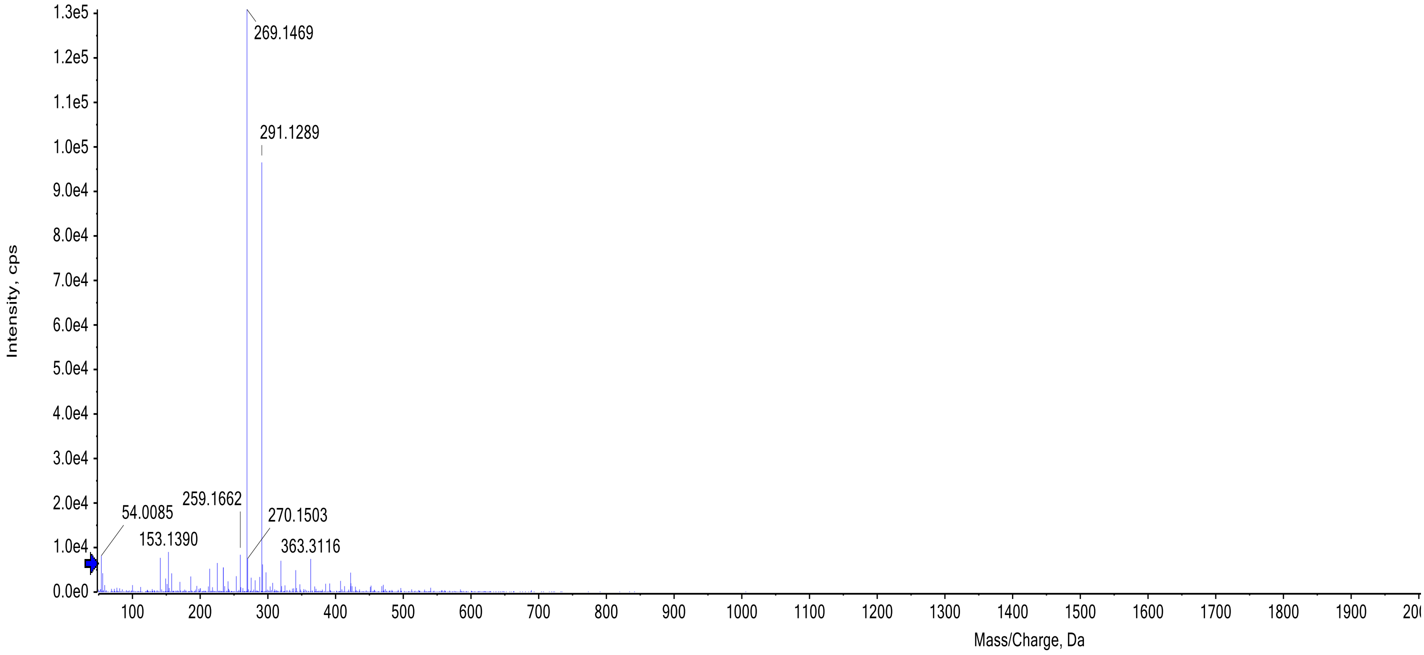
Intensity, cps
Mass/Charge, Da
269.1469
291.1289
259.1662
54.0085
153.1390
270.1503
363.3116

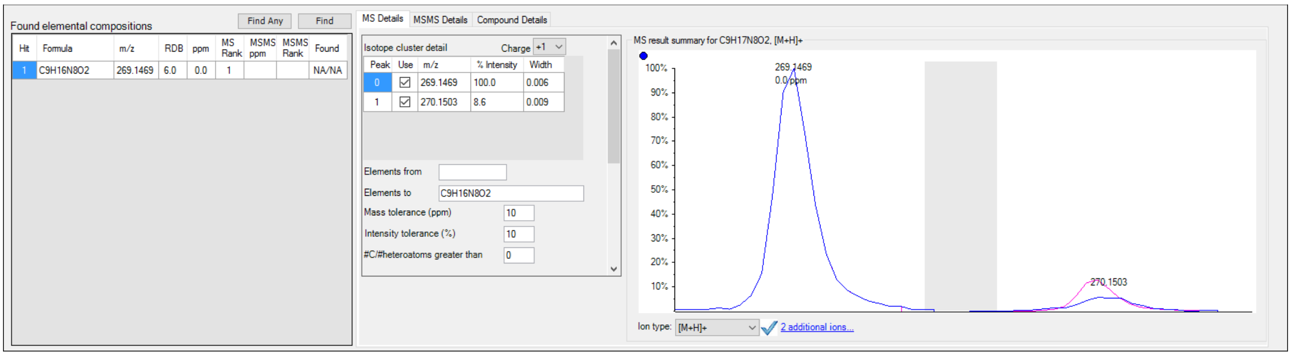
Found elemental compositions
Find Any
Find
Hit
Formula
m/z
RDB
ppm
MS Rank
MSMS ppm
MSMS Rank
Found
1
C9H16N8O2
269.1469
6.0
0.0
1
NA/NA
MS Details
MSMS Details
Compound Details
Isotope cluster detail
Charge +1
Peak
Use
m/z
% Intensity
Width
0
269.1469
100.0
0.006
1
270.1503
8.6
0.009
Elements from
Elements to
C9H16N8O2
Mass tolerance (ppm)
10
Intensity tolerance (%)
10
#C/#heteroatoms greater than
0
MS result summary for C9H17N8O2, [M+H]+
269.1469
0.0 ppm
270.1503
Ion type: [M+H]+
2 additional ions...

Compound 10

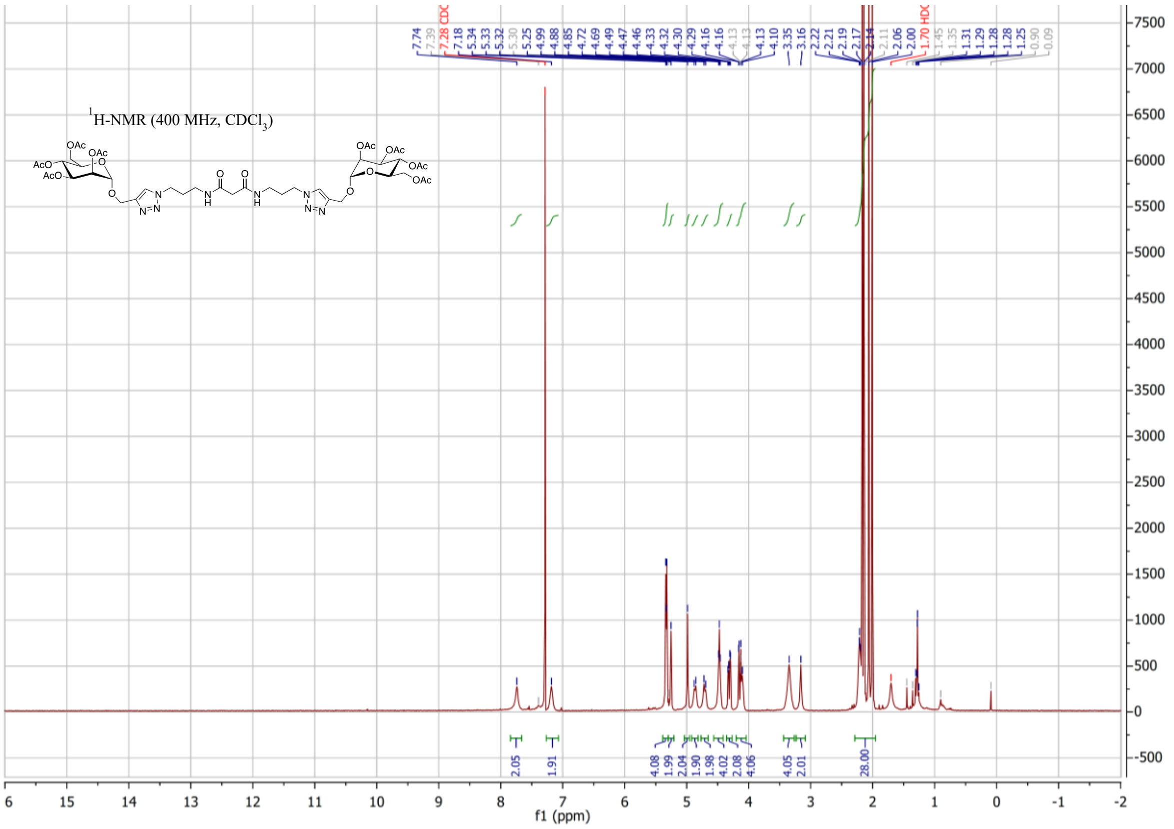

1H-NMR (400 MHz, CDCl3)
f1 (ppm)

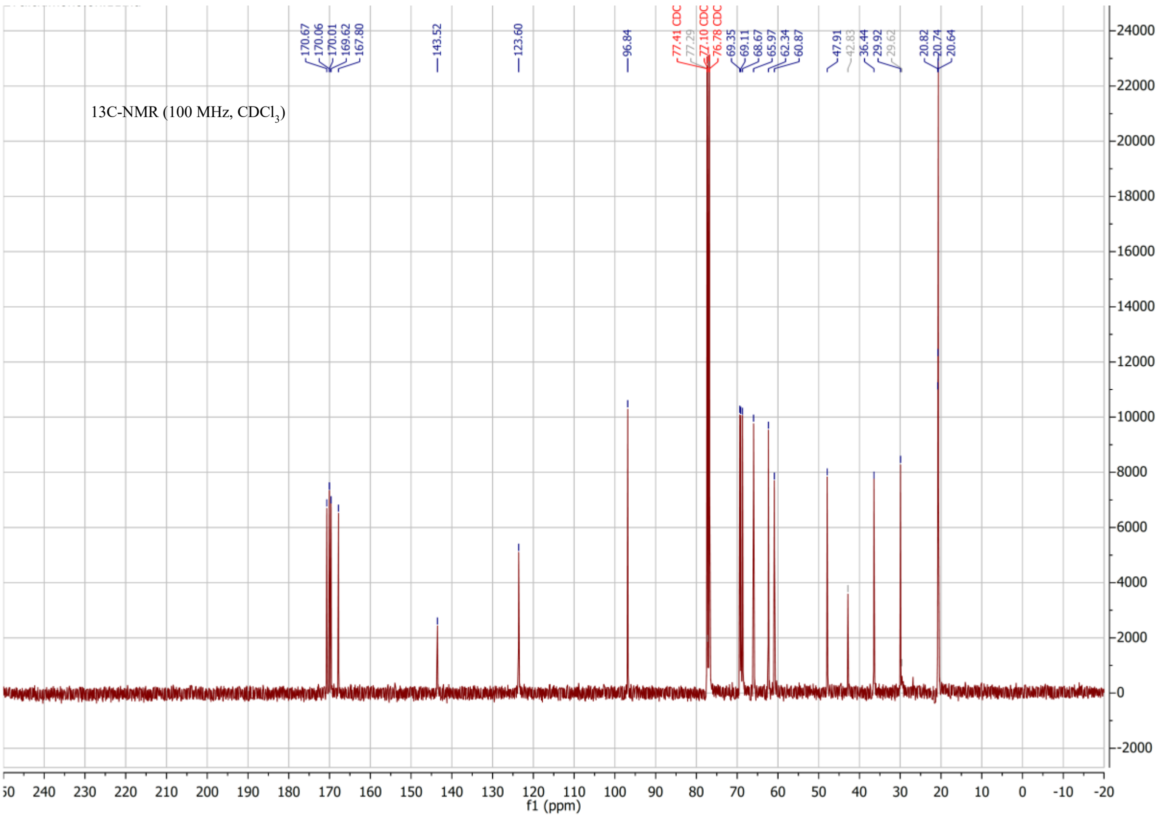

13C-NMR (100 MHz, CDCl3)
170.67
170.06
170.01
169.62
167.80
143.52
123.60
96.84
69.35
69.11
68.67
65.97
62.34
60.87
47.91
42.83
36.44
29.92
29.62
20.82
20.74
20.64
f1 (ppm)

Display Report

Analysis Info

Analysis Name	D:\Data\GOBS\Lisa\LT.click.mono.hs.d	Acquisition Date	10/8/2019 3:33:58 PM
Method	tune_high.m	Operator	BDAL@DE
Sample Name	LT.click.mono.hs	Instrument / Ser#	micrOTOF 235
Comment			

Acquisition Parameter

Source Type	ESI	Ion Polarity	Positive	Set Nebulizer	5.8 psi
Focus	Active			Set Dry Heater	180 °C
Scan Begin	50 m/z	Set Capillary	4500 V	Set Dry Gas	4.0 l/min
Scan End	3000 m/z	Set End Plate Offset	-500 V	Set Divert Valve	Waste

Meas. m/z	#	Formula	m/z	err [ppm]	mSigma	err [mDa]
1063.3716	1	C 43 H 60 N 8 Na O 22	1063.3714	-0.2	27.3	-0.2

Meas. m/z	#	Formula	m/z	err [ppm]	mSigma	err [mDa]

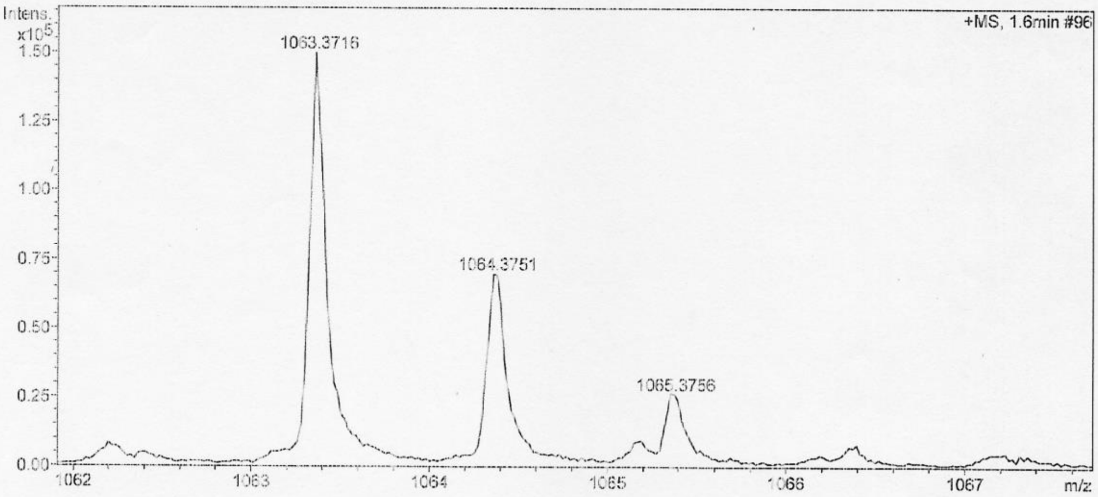

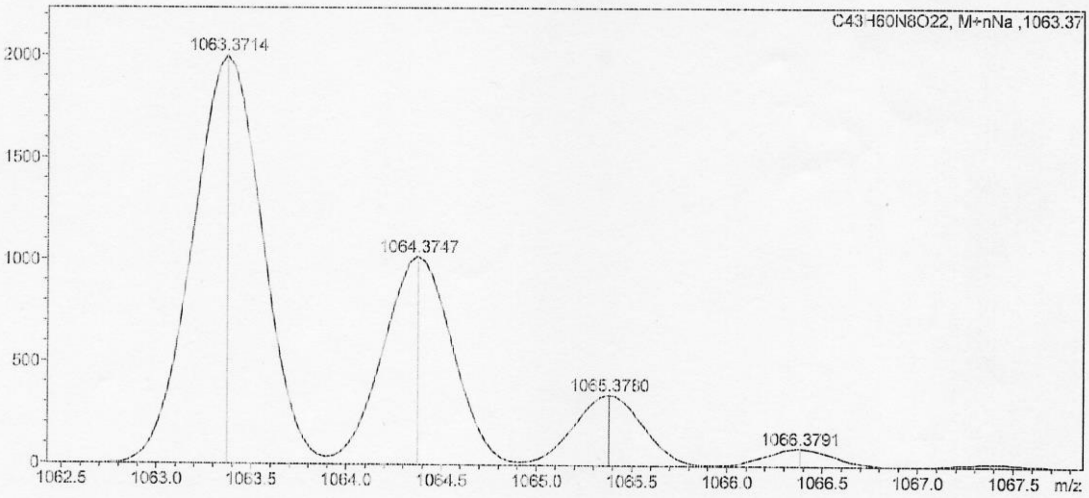

Compound 11

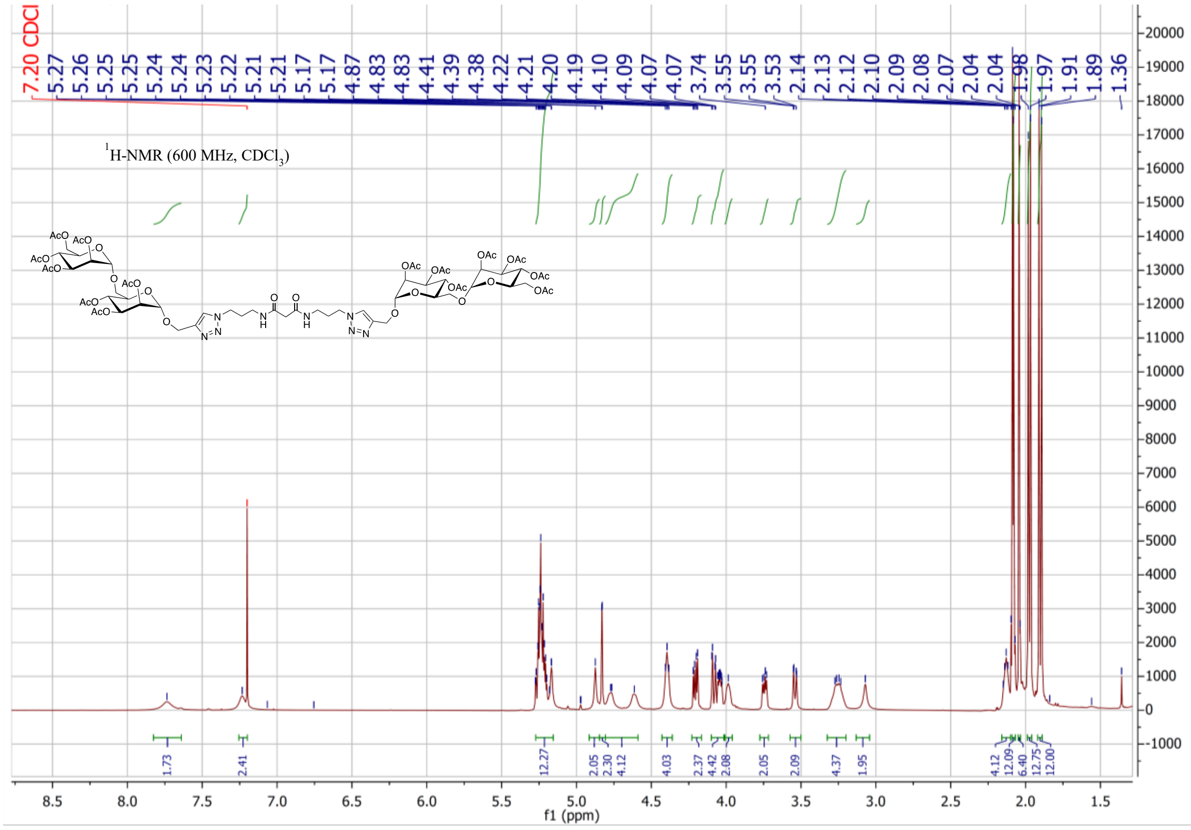

7.20 CDCl
5.27
5.26
5.25
5.25
5.24
5.24
5.23
5.22
5.21
5.21
5.17
5.17
4.87
4.83
4.41
4.39
4.38
4.22
4.21
4.20
4.19
4.10
4.09
4.07
4.07
3.74
3.55
3.55
3.53
2.14
2.13
2.12
2.10
2.09
2.08
2.07
2.04
2.04
1.98
1.97
1.91
1.89
1.36
^{1}H-NMR (600 MHz, $CDCl_3$)
f1 (ppm)

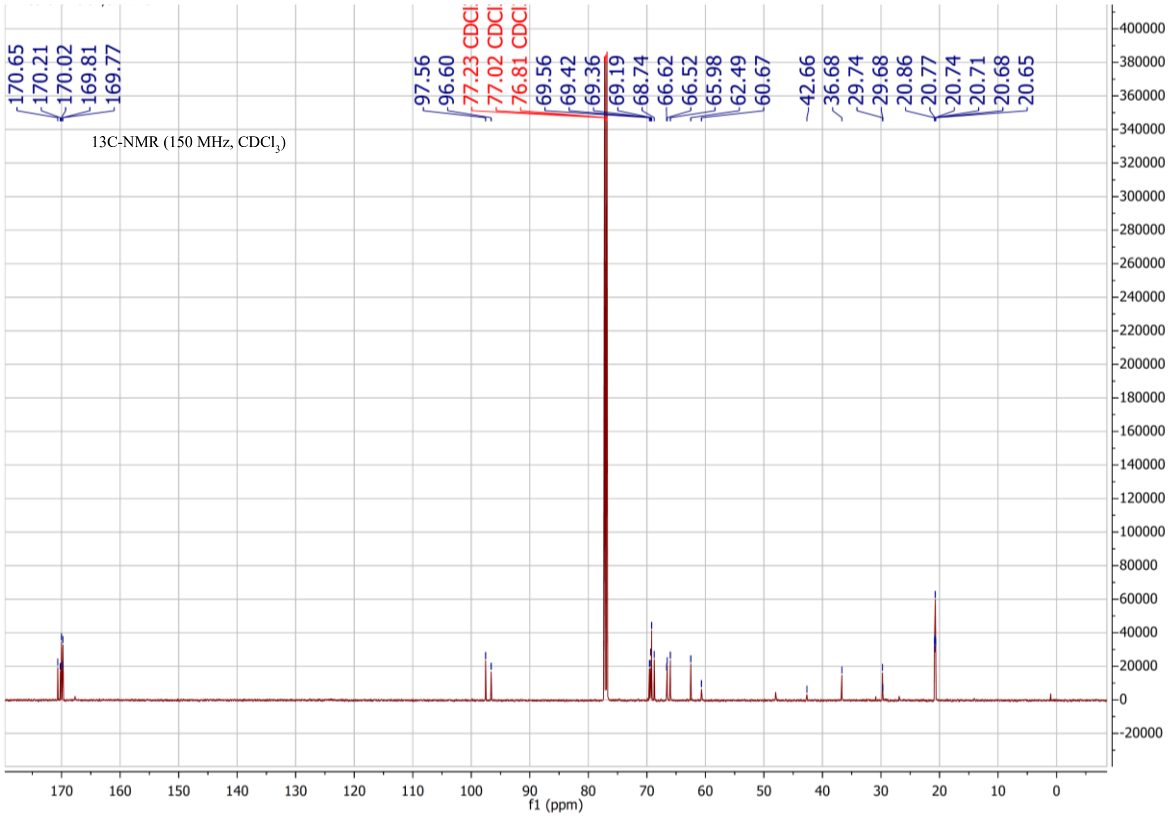

170.65
170.21
170.02
169.81
169.77
97.56
96.60
77.23 CDCl
77.02 CDCl
76.81 CDCl
69.56
69.42
69.36
69.19
68.74
66.62
66.52
65.98
62.49
60.67
42.66
36.68
29.74
29.68
20.86
20.77
20.74
20.71
20.68
20.65
13C-NMR (150 MHz, $CDCl_3$)
f1 (ppm)

Display Report

Analysis Info

Analysis Name	D:\Data\GOBS\Lisa\LT click dis 1,6 HRMS 4.d	Acquisition Date	11/25/2020 11:26:45 AM
Method	tune_high.m	Operator	BDAL@DE
Sample Name	LT click dis 1,6 HRMS 4	Instrument / Ser#	micrOTOF 235
Comment			

Acquisition Parameter

Source Type	ESI	Ion Polarity	Positive	Set Nebulizer	5.8 psi
Focus	Active			Set Dry Heater	180 °C
Scan Begin	50 m/z	Set Capillary	4500 V	Set Dry Gas	4.0 l/min
Scan End	4000 m/z	Set End Plate Offset	-500 V	Set Divert Valve	Source

Meas. m/z	z	#	Formula	m/z	err [ppm]	mSigma	err [mDa]

Meas. m/z	z	#	Formula	m/z	err [ppm]	mSigma	err [mDa]
1639.5405	1+	1	C 67 H 92 N 8 Na O 38	1639.5405	-0.0	8.7	-0.0

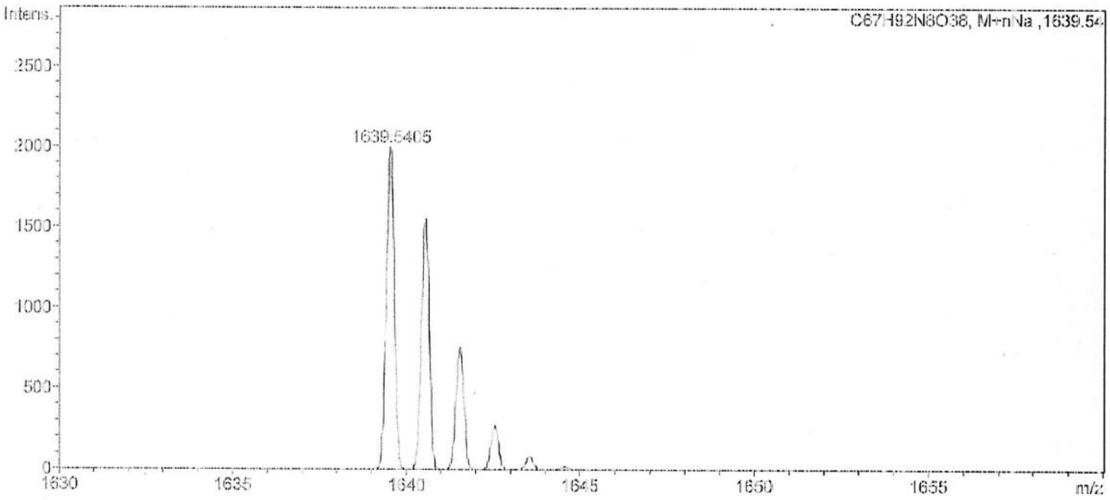

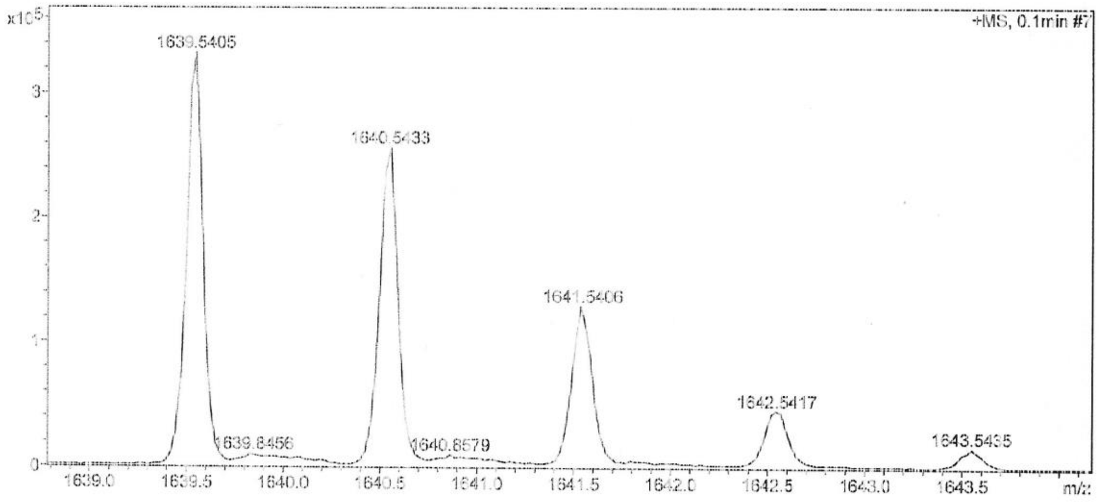

Bruker Compass DataAnalysis 4.0 printed: 11/25/2020 11:36:34 AM Page 1 of 1

Compound 12

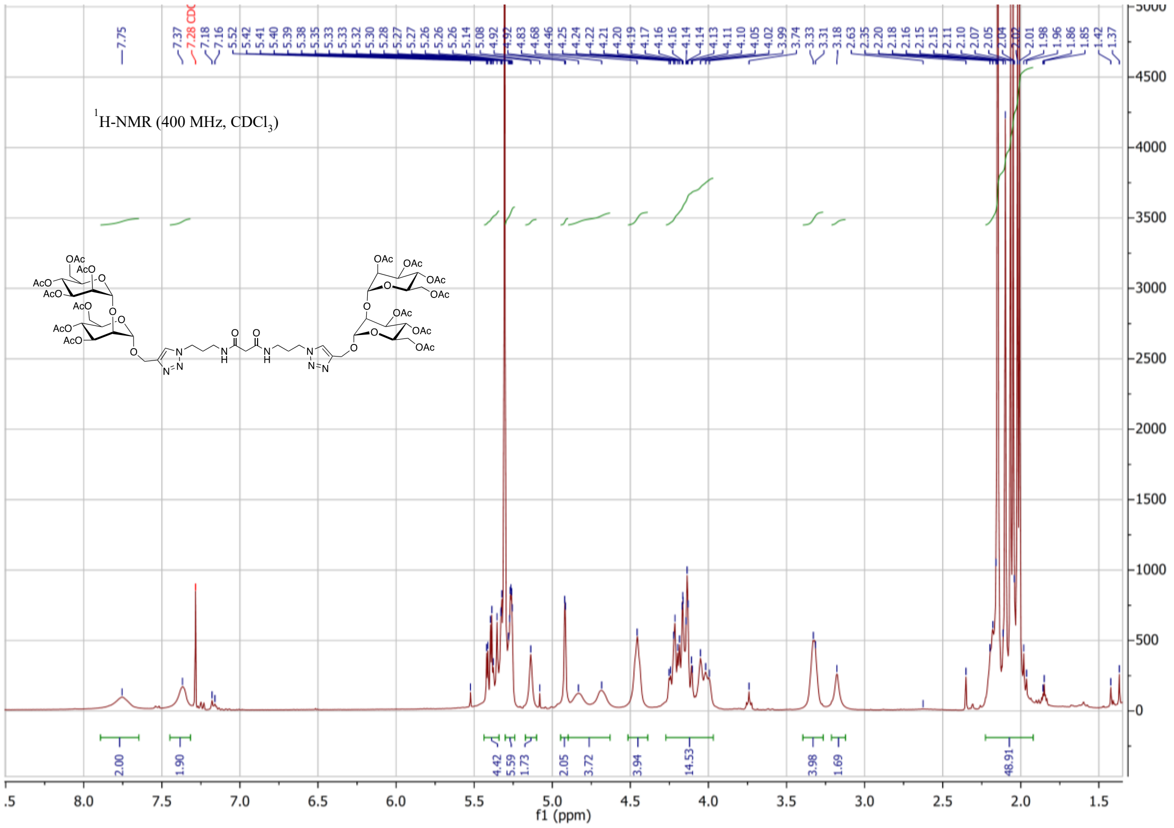

1H-NMR (400 MHz, CDCl3)
7.75
7.37
7.28 CDC
7.18
7.16
5.52
5.42
5.41
5.40
5.39
5.38
5.35
5.33
5.33
5.32
5.30
5.28
5.27
5.27
5.26
5.26
5.26
5.14
5.08
4.92
4.92
4.83
4.68
4.46
4.25
4.24
4.22
4.21
4.20
4.19
4.17
4.16
4.14
4.14
4.13
4.11
4.10
4.05
4.02
3.99
3.74
3.33
3.31
3.18
2.63
2.35
2.20
2.18
2.16
2.15
2.15
2.11
2.10
2.07
2.05
2.04
2.02
2.01
1.98
1.96
1.86
1.85
1.42
1.37
2.00
1.90
4.42
5.59
1.73
2.05
3.72
3.94
14.53
3.98
1.69
48.91
f1 (ppm)

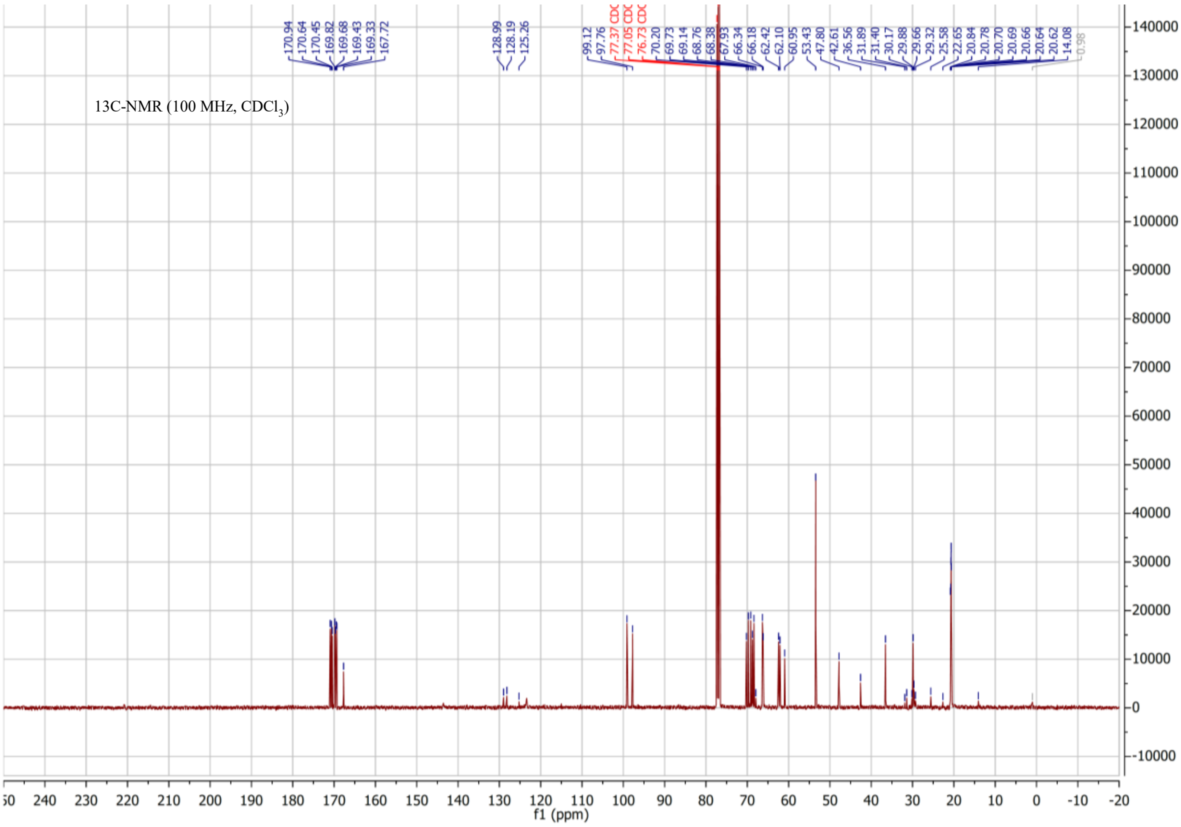

13C-NMR (100 MHz, CDCl3)
170.94
170.64
170.45
169.82
169.68
169.43
169.33
167.72
128.99
128.19
125.26
99.12
97.76
77.37 CDC
77.05 CDC
76.73 CDC
70.20
69.73
69.14
68.76
68.38
67.93
66.34
66.18
62.42
62.10
60.95
53.43
47.80
42.61
36.56
31.89
31.40
30.17
29.88
29.66
29.32
25.58
22.65
20.84
20.78
20.70
20.69
20.66
20.64
20.62
14.08
0.98
f1 (ppm)

Display Report

Analysis Info

Analysis Name	D:\Data\GOBS\Lisa\LT.click.dis.1.2hs .d	Acquisition Date	10/9/2019 4:45:18 PM
Method	tune_high.m	Operator	BDAL@DE
Sample Name	LT.click.dis.1.2hs	Instrument / Ser#	micrOTOF 235
Comment			

Acquisition Parameter

Source Type	ESI	Ion Polarity	Positive	Set Nebulizer	5.8 psi
Focus	Active			Set Dry Heater	180 °C
Scan Begin	50 m/z	Set Capillary	4500 V	Set Dry Gas	4.0 l/min
Scan End	3000 m/z	Set End Plate Offset	-500 V	Set Divert Valve	Waste

Meas. m/z	#	Formula	m/z	err [ppm]	mSigma	err [mDa]
1639.5405	1	C 67 H 92 N 8 Na O 38	1639.5405	-0.0	20.6	-0.0

Meas. m/z	#	Formula	m/z	err [ppm]	mSigma	err [mDa]

Meas. m/z	#	Formula	m/z	err [ppm]	mSigma	err [mDa]

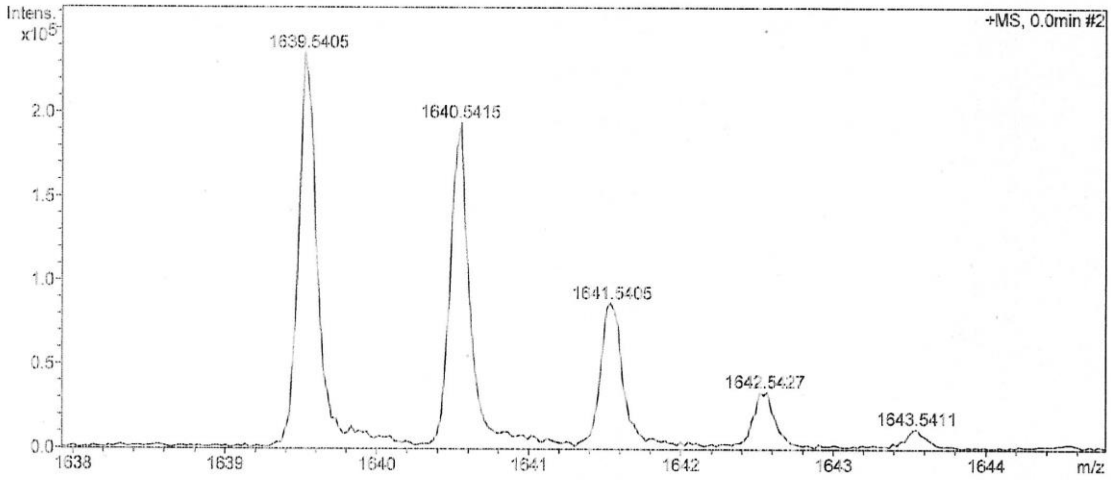

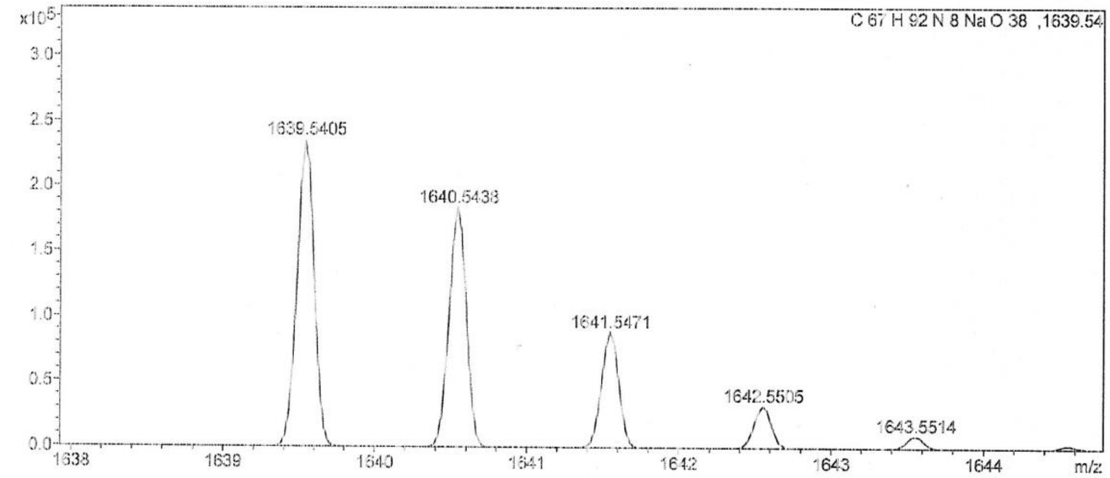

Bruker Compass DataAnalysis 4.0 printed: 10/9/2019 4:54:58 PM Page 1 of 1

Compound 13

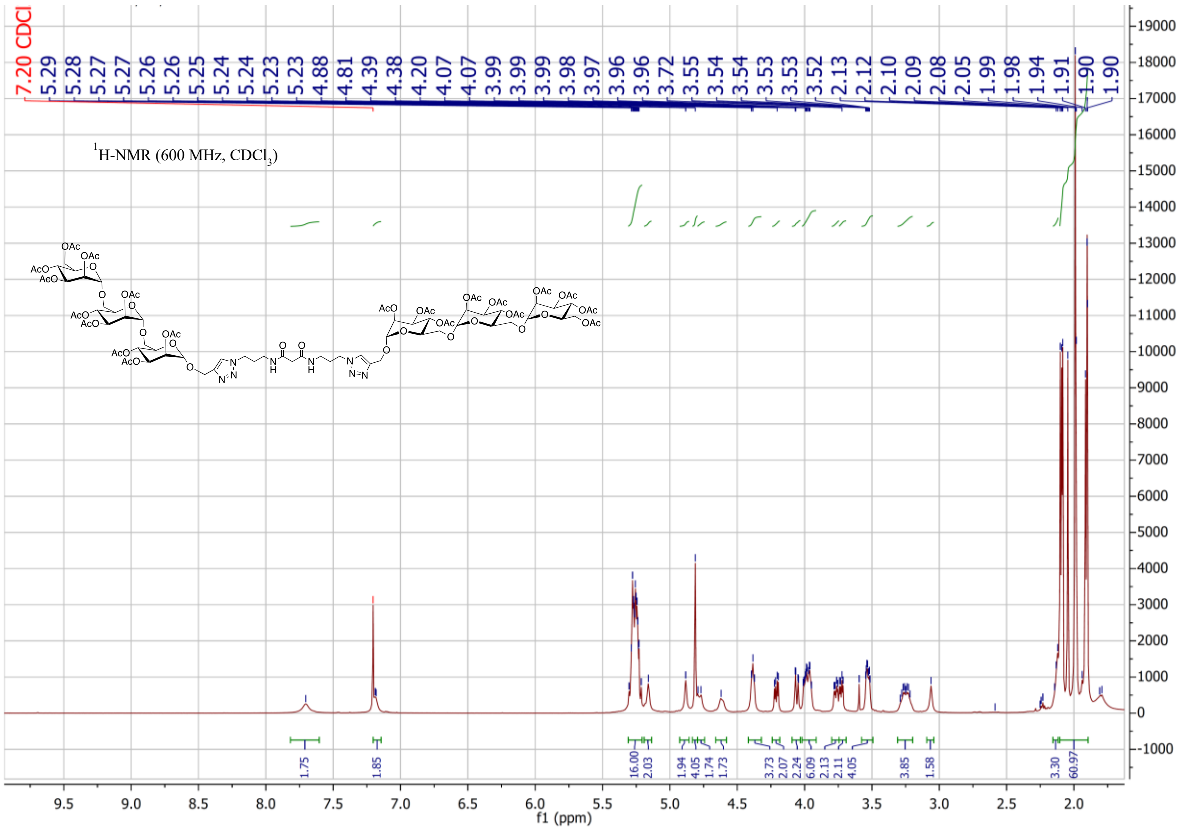
7.20 CDCl
5.29 5.28 5.27 5.27 5.26 5.26 5.25 5.24 5.24 5.23 5.23 4.88 4.81 4.39 4.38 4.20 4.07 4.07 3.99 3.99 3.99 3.98 3.97 3.96 3.96 3.72 3.55 3.54 3.54 3.53 3.53 3.52 2.13 2.12 2.10 2.09 2.08 2.05 1.99 1.98 1.94 1.91 1.90 1.90
1H-NMR (600 MHz, CDCl3)
1.75 1.85 16.00 2.03 1.94 4.05 1.74 1.73 3.73 2.07 2.24 6.09 2.13 2.11 4.05 3.85 1.58 3.30 60.97
f1 (ppm)

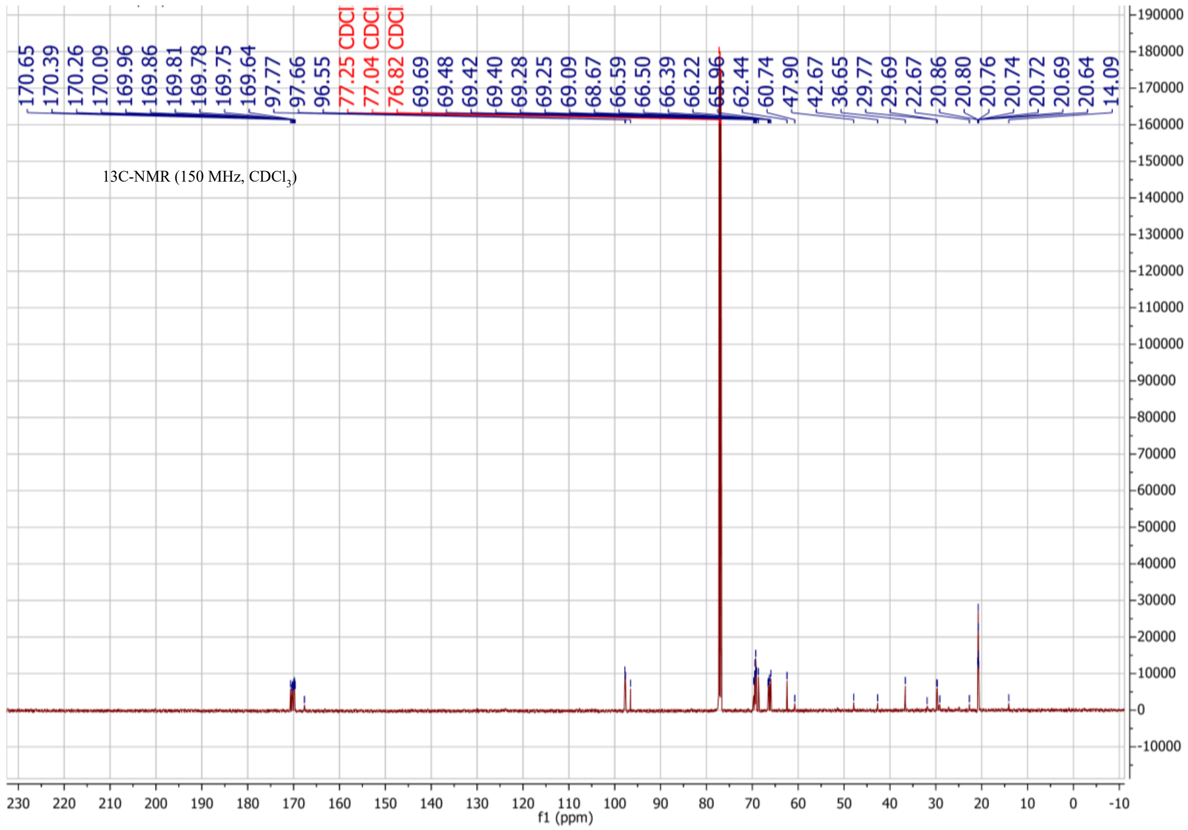
170.65 170.39 170.26 170.09 169.96 169.86 169.81 169.78 169.75 169.64 97.77 97.66 96.55
77.25 CDCl 77.04 CDCl 76.82 CDCl
69.69 69.48 69.42 69.40 69.28 69.25 69.09 68.67 66.59 66.50 66.39 66.22 65.96 62.44 60.74 47.90 42.67 36.65 29.77 29.69 22.67 20.86 20.80 20.76 20.74 20.72 20.69 20.64 14.09
13C-NMR (150 MHz, CDCl3)
f1 (ppm)

Display Report

Analysis Info

Analysis Name	D:\Data\GOBS\Lisa\LT.click.tris.1.6.1.6.hs .d	Acquisition Date	10/9/2019 4:57:05 PM
Method	tune_high.m	Operator	BDAL@DE
Sample Name	LT.click.tris.1.6.1.6.hs	Instrument / Ser#	micrOTOF 235
Comment			

Acquisition Parameter

Source Type	ESI	Ion Polarity	Positive	Set Nebulizer	5.8 psi
Focus	Active			Set Dry Heater	180 °C
Scan Begin	50 m/z	Set Capillary	4500 V	Set Dry Gas	4.0 l/min
Scan End	3000 m/z	Set End Plate Offset	-500 V	Set Divert Valve	Waste

Meas. m/z	#	Formula	m/z	err [ppm]	mSigma	err [mDa]
2216.7143	1	C 91 H 125 N 8 Na O 54	2216.7173	1.4	215.9	3.1

Meas. m/z	#	Formula	m/z	err [ppm]	mSigma	err [mDa]

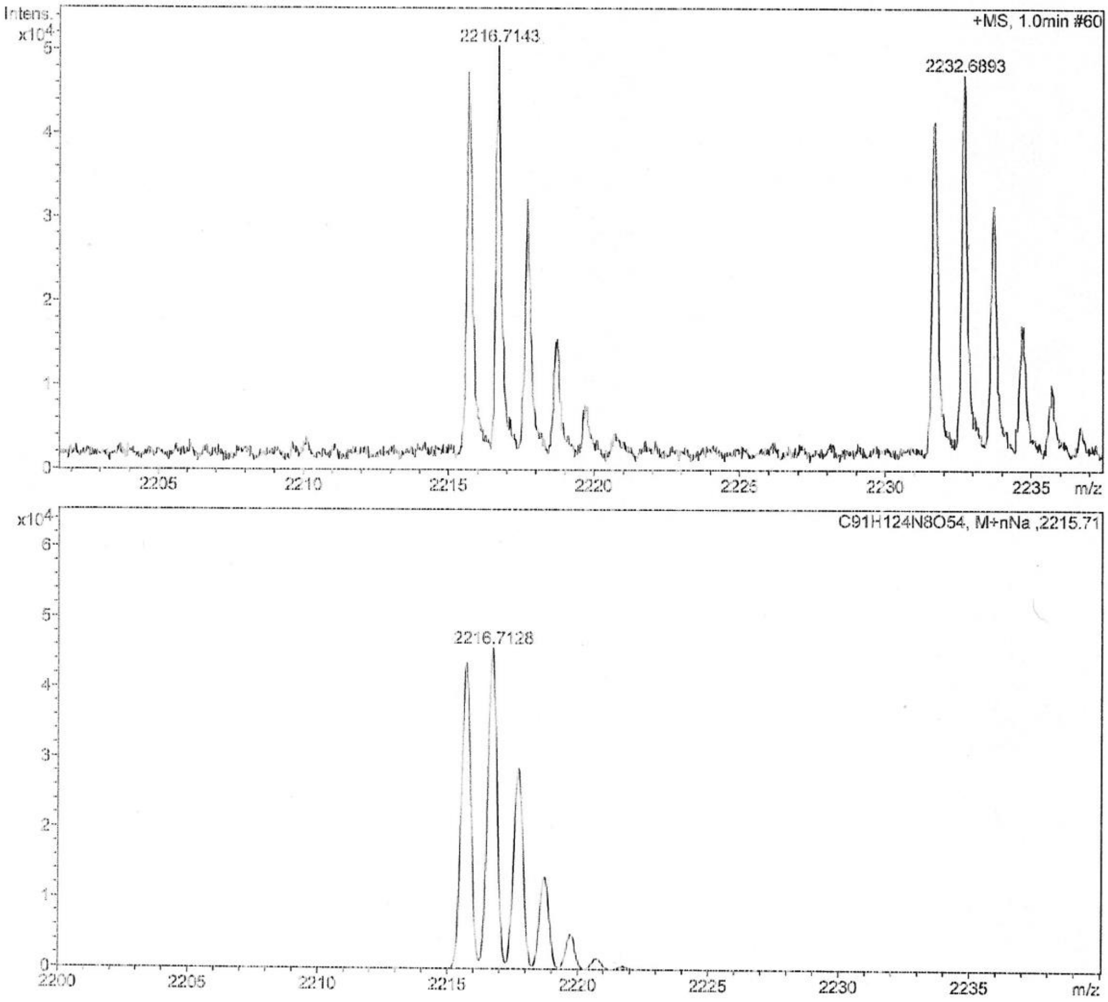

Compound 14

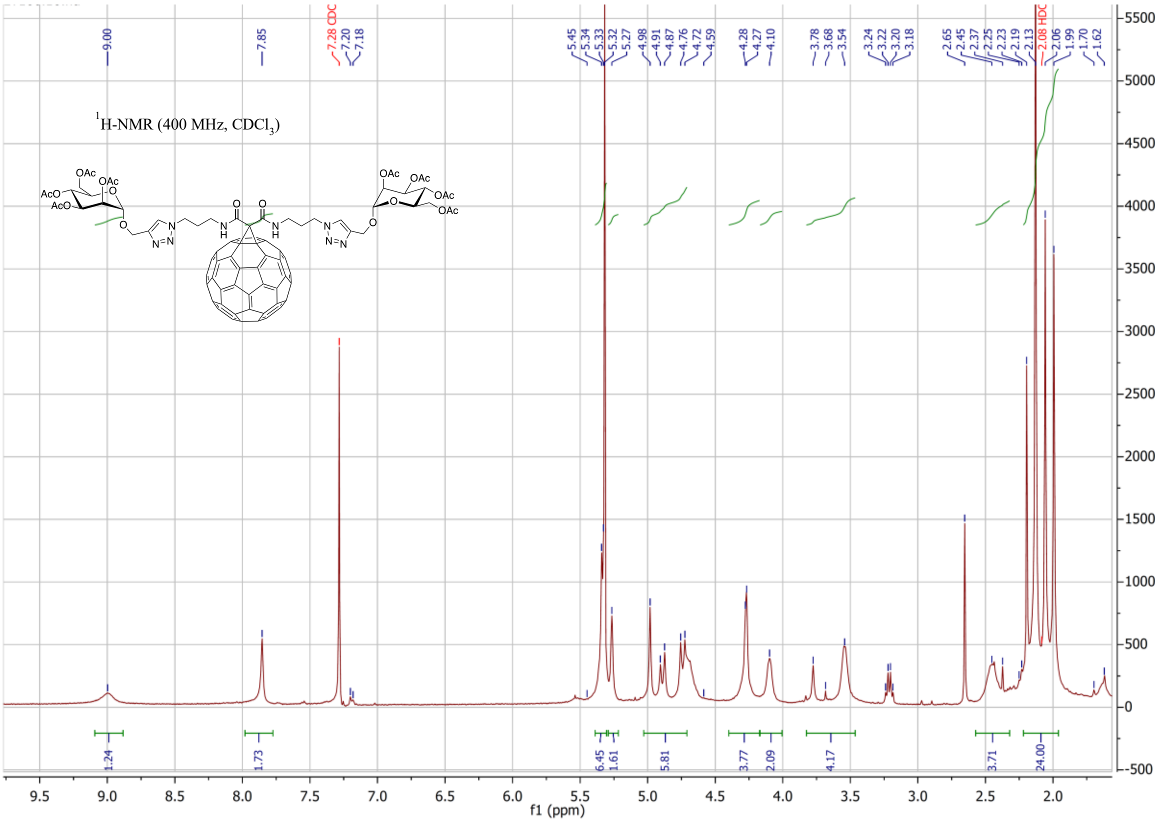

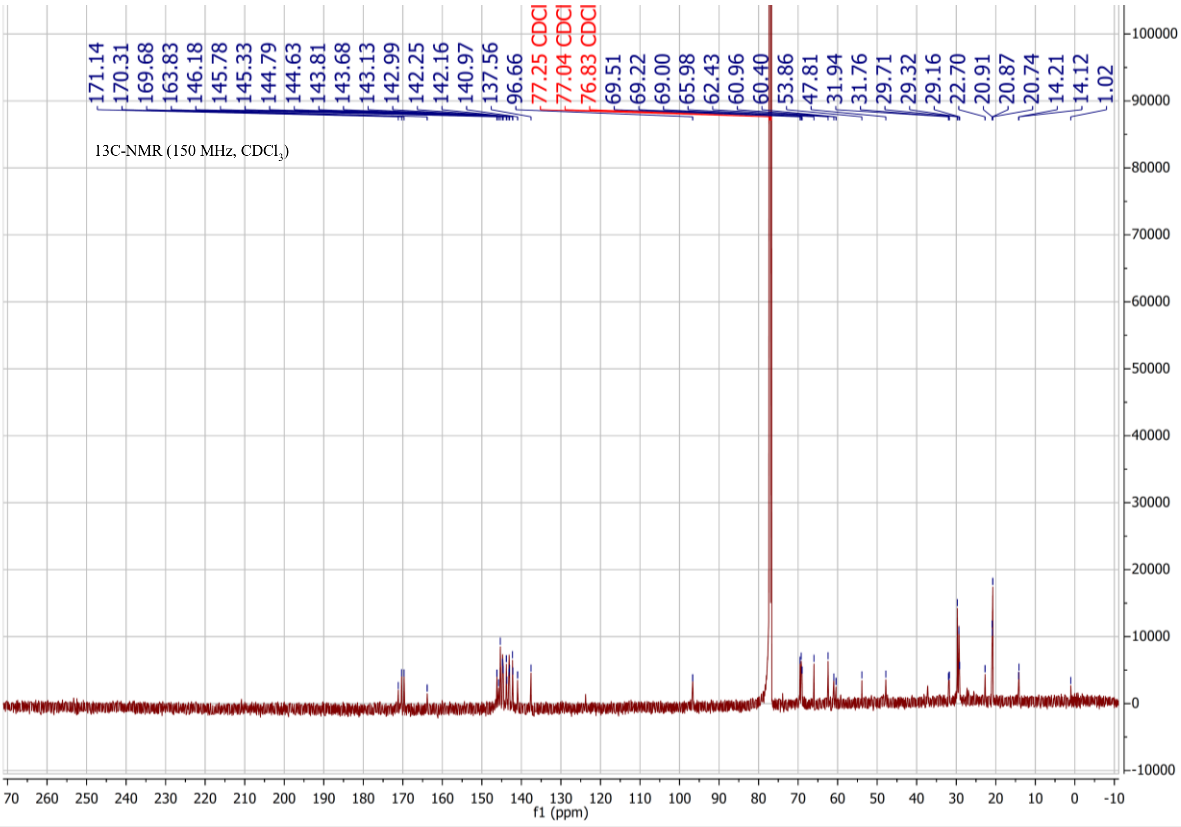

Display Report

Analysis Info

Analysis Name	D:\Data\GOBS\Lisa\LT c60 mono hrms.d
Method	tune_high.m
Sample Name	LT c60 mono hrms
Comment	
Acquisition Date	11/20/2020 5:35:25 PM
Operator	BDAL@DE
Instrument / Ser#	micrOTOF 235

Acquisition Parameter

Source Type	ESI	Ion Polarity	Positive	Set Nebulizer	5.8 psi
Focus	Active			Set Dry Heater	180 °C
Scan Begin	50 m/z	Set Capillary	4500 V	Set Dry Gas	4.0 l/min
Scan End	4000 m/z	Set End Plate Offset	-500 V	Set Divert Valve	Source

Meas. m/z	z	#	Formula	m/z	err [ppm]	mSigma	err [mDa]
1782.3555	1+	1	C 103 H 58 N 8 Na O 22	1781.3558	1.8	33.4	3.3

Meas. m/z	z	#	Formula	m/z	err [ppm]	mSigma	err [mDa]

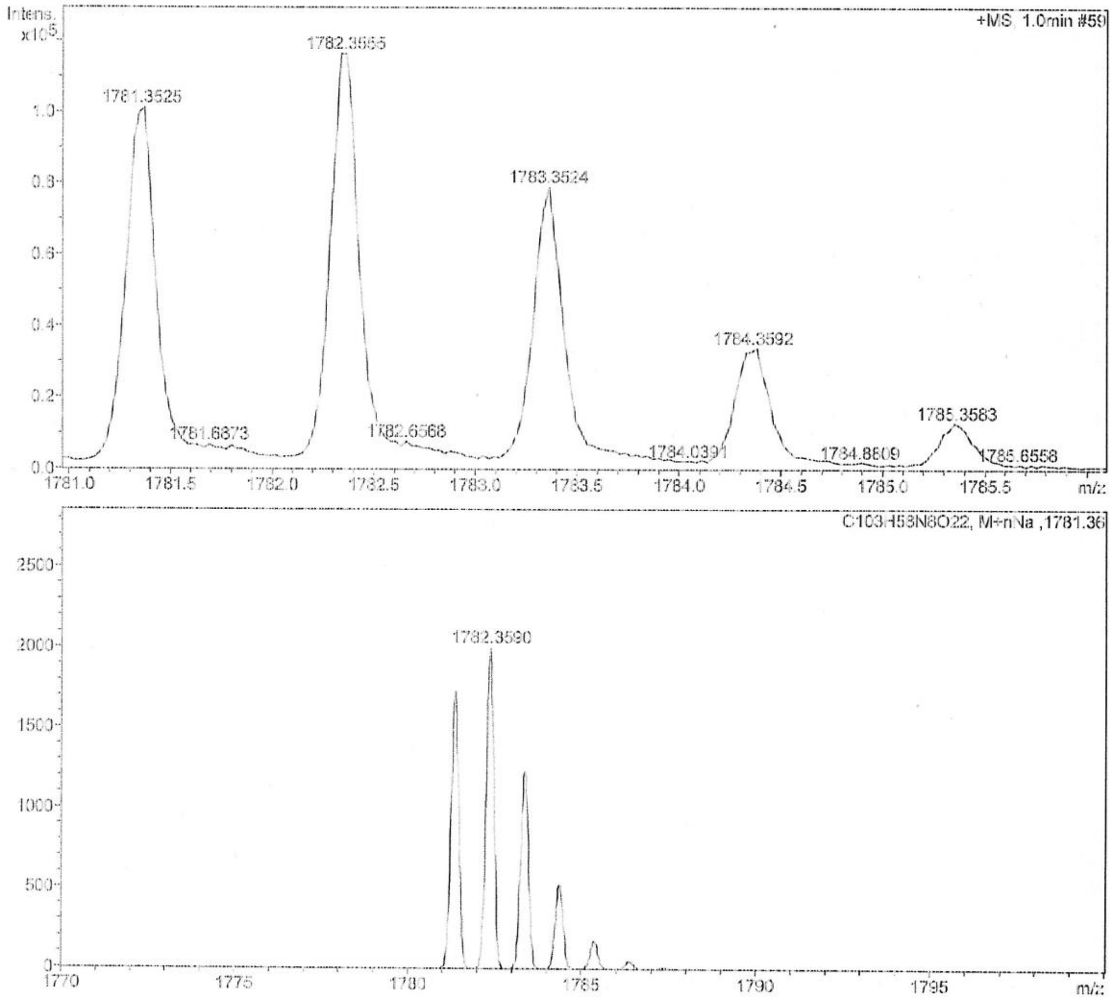

Bruker Compass DataAnalysis 4.0 printed: 11/25/2020 9:35:14 AM Page 1 of 1

Compound 15

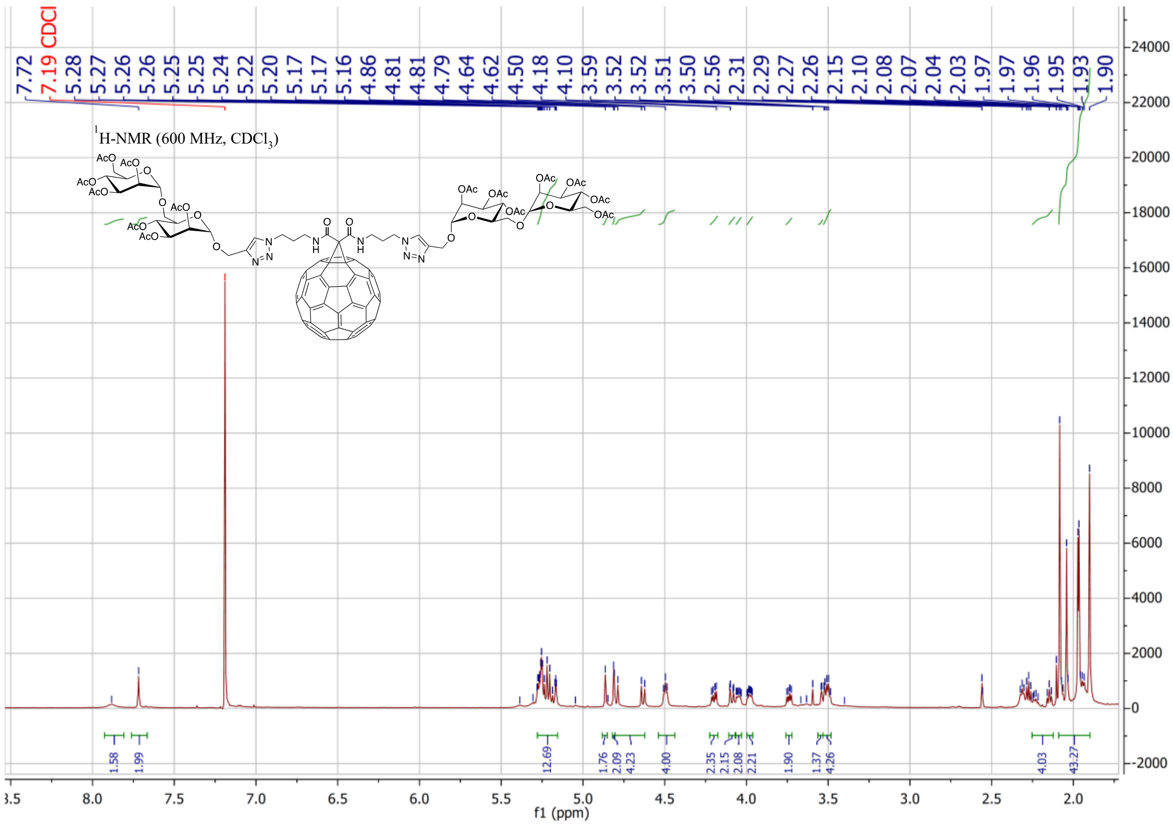

7.72
7.19 CDCl
5.28
5.27
5.26
5.26
5.25
5.25
5.24
5.22
5.20
5.17
5.17
5.16
4.86
4.81
4.81
4.79
4.64
4.62
4.50
4.18
4.10
3.59
3.52
3.52
3.51
3.50
2.56
2.31
2.29
2.27
2.26
2.15
2.10
2.08
2.07
2.04
2.03
1.97
1.97
1.96
1.95
1.93
1.90
^{1}H-NMR (600 MHz, $CDCl_3$)
f1 (ppm)

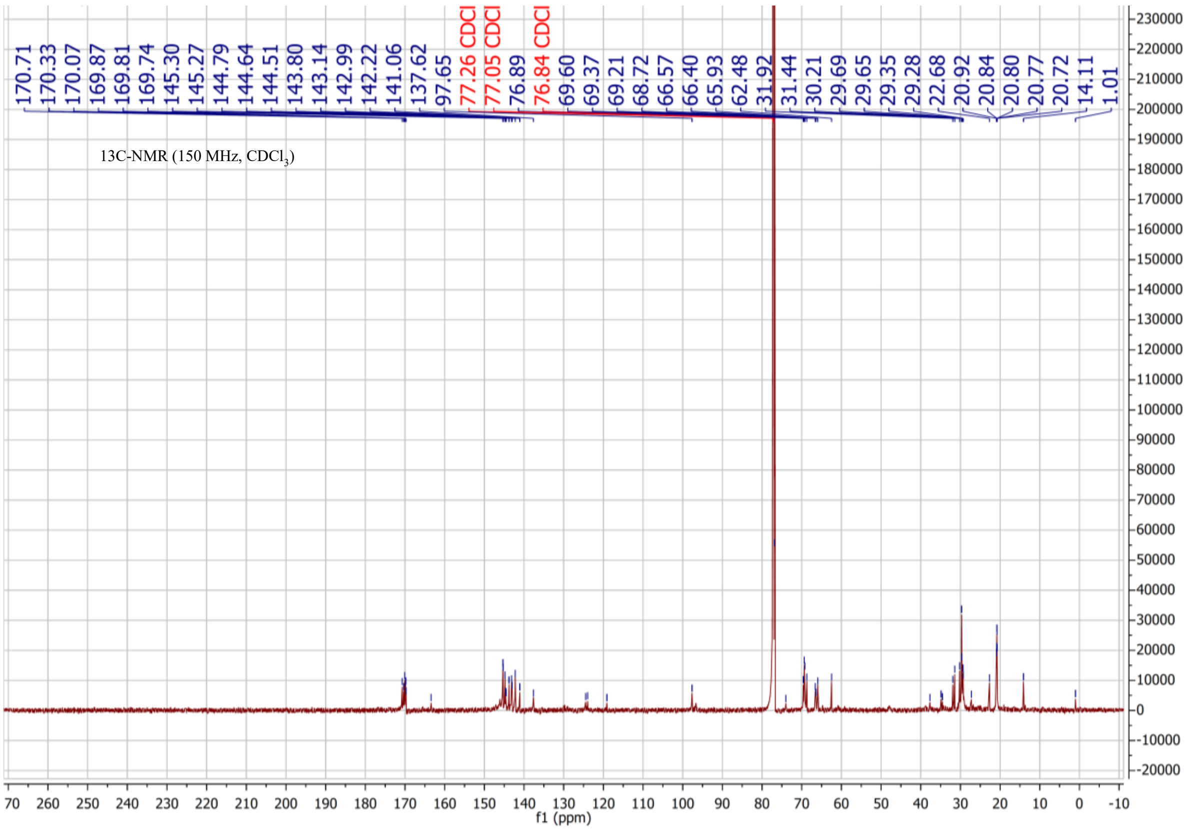

170.71
170.33
170.07
169.87
169.81
169.74
145.30
145.27
144.79
144.64
144.51
143.80
143.14
142.99
142.22
141.06
137.62
97.65
77.26 CDCl
77.05 CDCl
76.89
76.84 CDCl
69.60
69.37
69.21
68.72
66.57
66.40
65.93
62.48
31.92
31.44
30.21
29.69
29.65
29.35
29.28
22.68
20.92
20.84
20.80
20.77
20.72
14.11
1.01
13C-NMR (150 MHz, $CDCl_3$)
f1 (ppm)

Display Report

Analysis Info

Analysis Name	D:\Data\GOBS\Lisa\LT click dis 1,6 HRMS 4.d	Acquisition Date	11/25/2020 11:26:45 AM
Method	tune_high.m	Operator	BDAL@DE
Sample Name	LT click dis 1,6 HRMS 4	Instrument / Ser#	micrOTOF 235
Comment			

Acquisition Parameter

Source Type	ESI	Ion Polarity	Positive	Set Nebulizer	5.8 psi
Focus	Active			Set Dry Heater	180 °C
Scan Begin	50 m/z	Set Capillary	4500 V	Set Dry Gas	4.0 l/min
Scan End	4000 m/z	Set End Plate Offset	-500 V	Set Divert Valve	Source

Meas. m/z	z	#	Formula	m/z	err [ppm]	mSigma	err [mDa]

Meas. m/z	z	#	Formula	m/z	err [ppm]	mSigma	err [mDa]
1639.5405	1+	1	C 67 H 92 N 8 Na O 38	1639.5405	-0.0	8.7	-0.0

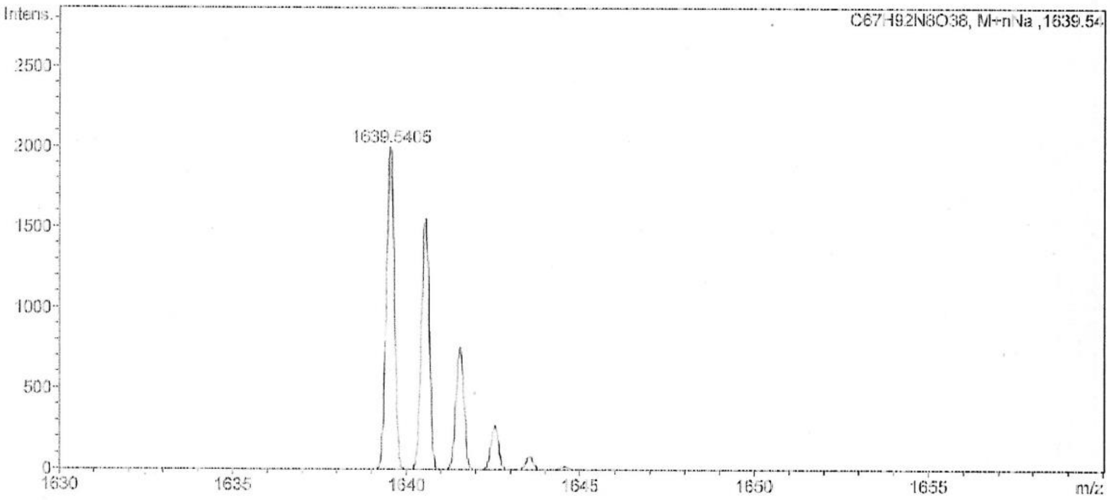

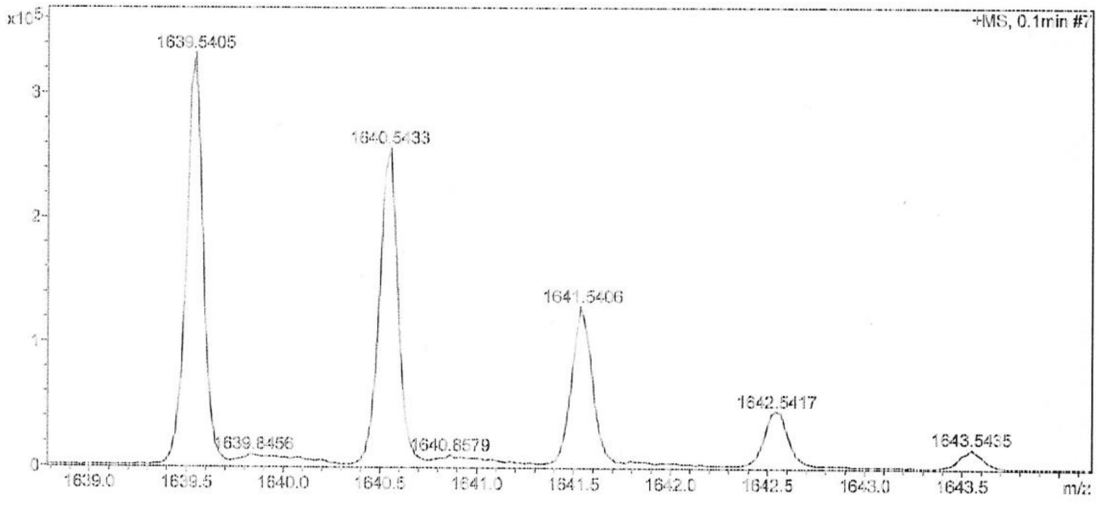

Bruker Compass DataAnalysis 4.0 printed: 11/25/2020 11:36:34 AM Page 1 of 1

Compound 16

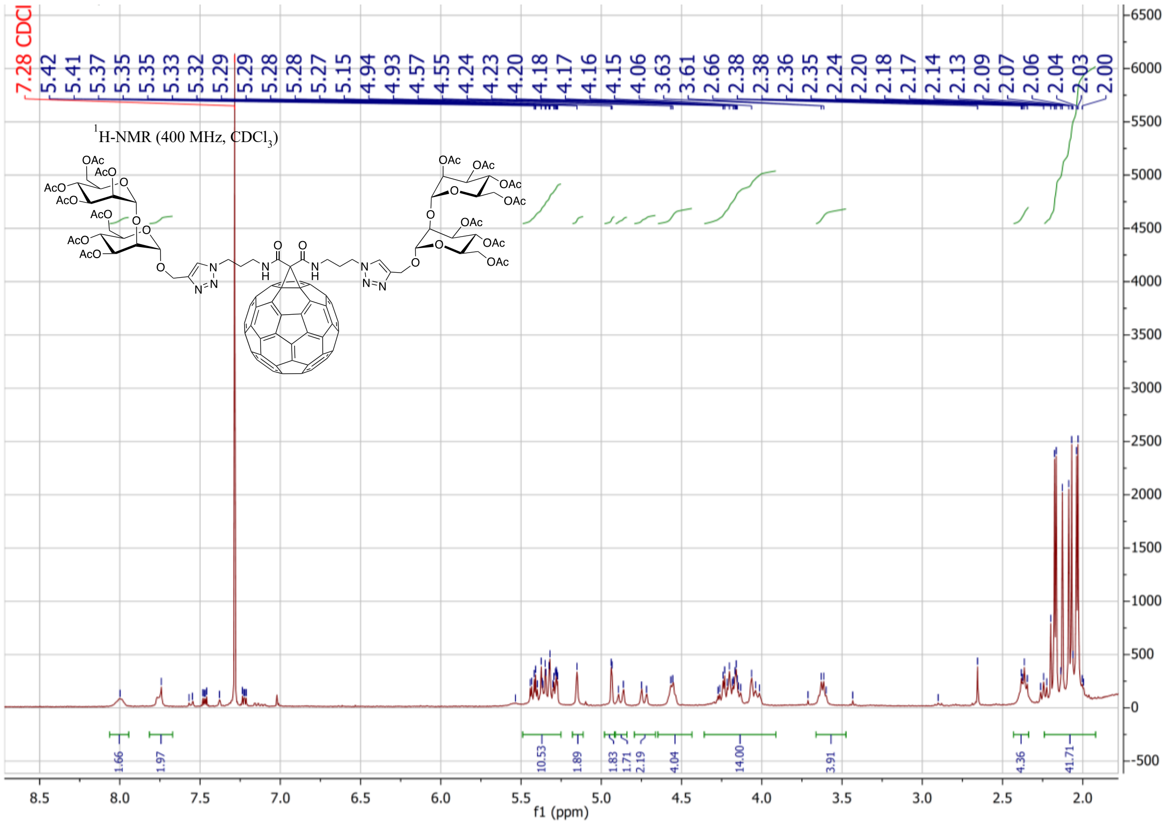
1H-NMR (400 MHz, $CDCl_3$)
7.28 CDCl
5.42
5.41
5.37
5.35
5.35
5.33
5.32
5.29
5.29
5.28
5.28
5.27
5.15
4.94
4.93
4.57
4.55
4.24
4.23
4.20
4.18
4.17
4.16
4.15
4.06
3.63
3.61
2.66
2.38
2.38
2.36
2.35
2.24
2.20
2.18
2.17
2.14
2.13
2.09
2.07
2.06
2.04
2.03
2.00
1.66
1.97
10.53
1.89
1.83
1.71
2.19
4.04
14.00
3.91
4.36
41.71
f1 (ppm)

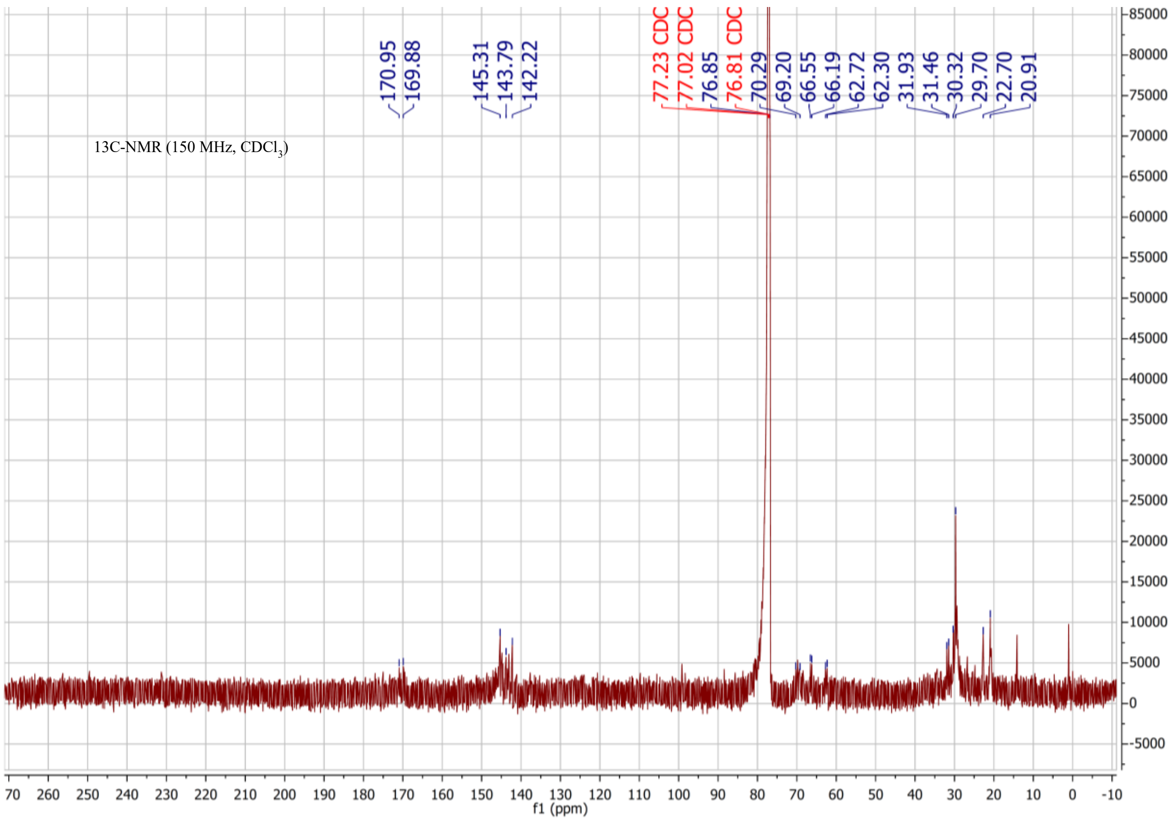
13C-NMR (150 MHz, $CDCl_3$)
170.95
169.88
145.31
143.79
142.22
77.23 CDC
77.02 CDC
76.85
76.81 CDC
70.29
69.20
66.55
66.19
62.72
62.30
31.93
31.46
30.32
29.70
22.70
20.91
f1 (ppm)

Display Report

Analysis Info

Analysis Name	D:\Data\GOBS\Lisa\LT.click.dis.1.2hs .d	Acquisition Date	10/9/2019 4:45:18 PM
Method	tune_high.m	Operator	BDAL@DE
Sample Name	LT.click.dis.1.2hs	Instrument / Ser#	micrOTOF 235
Comment			

Acquisition Parameter

Source Type	ESI	Ion Polarity	Positive	Set Nebulizer	5.8 psi
Focus	Active			Set Dry Heater	180 °C
Scan Begin	50 m/z	Set Capillary	4500 V	Set Dry Gas	4.0 l/min
Scan End	3000 m/z	Set End Plate Offset	-500 V	Set Divert Valve	Waste

Meas. m/z	#	Formula	m/z	err [ppm]	mSigma	err [mDa]
1639.5405	1	C 67 H 92 N 8 Na O 38	1639.5405	-0.0	20.6	-0.0

Meas. m/z	#	Formula	m/z	err [ppm]	mSigma	err [mDa]

Meas. m/z	#	Formula	m/z	err [ppm]	mSigma	err [mDa]

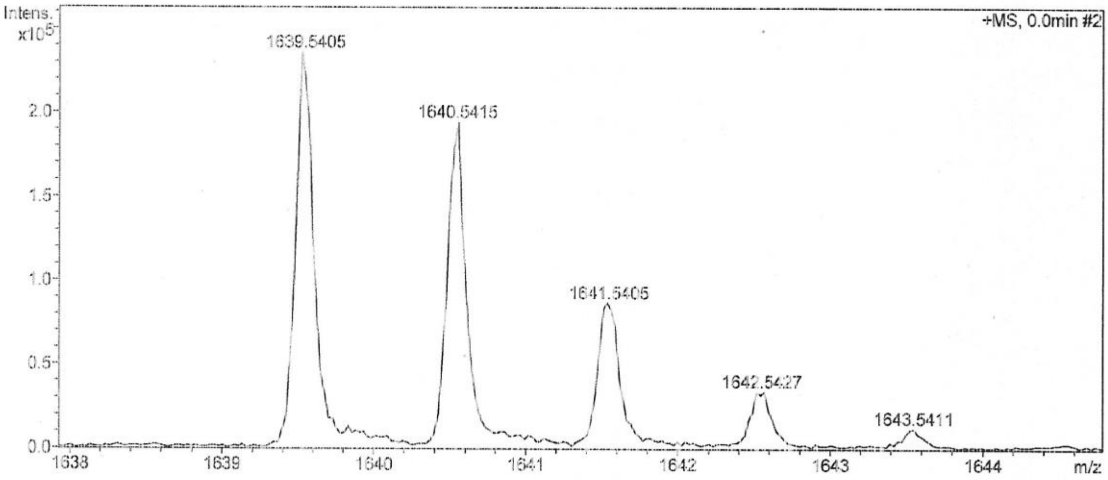

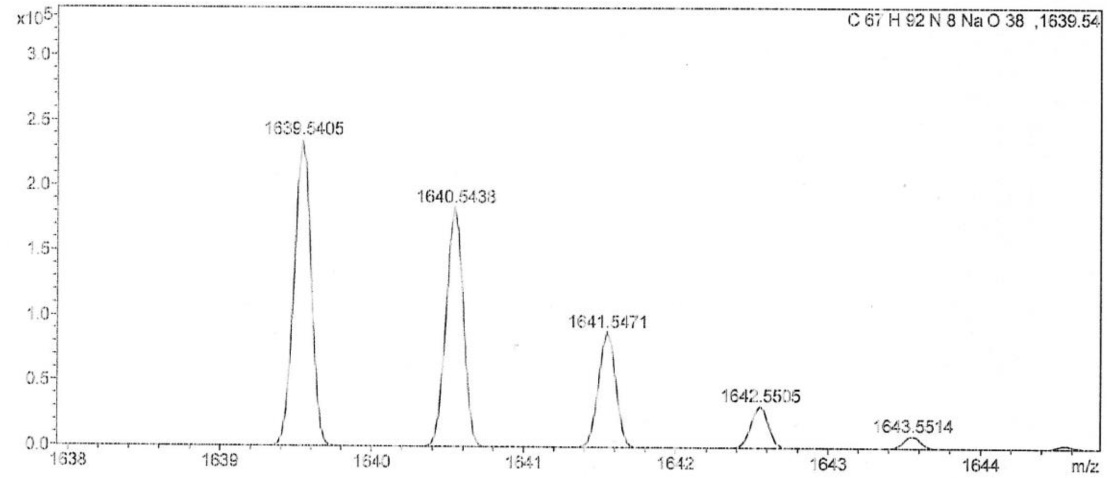

Compound 17

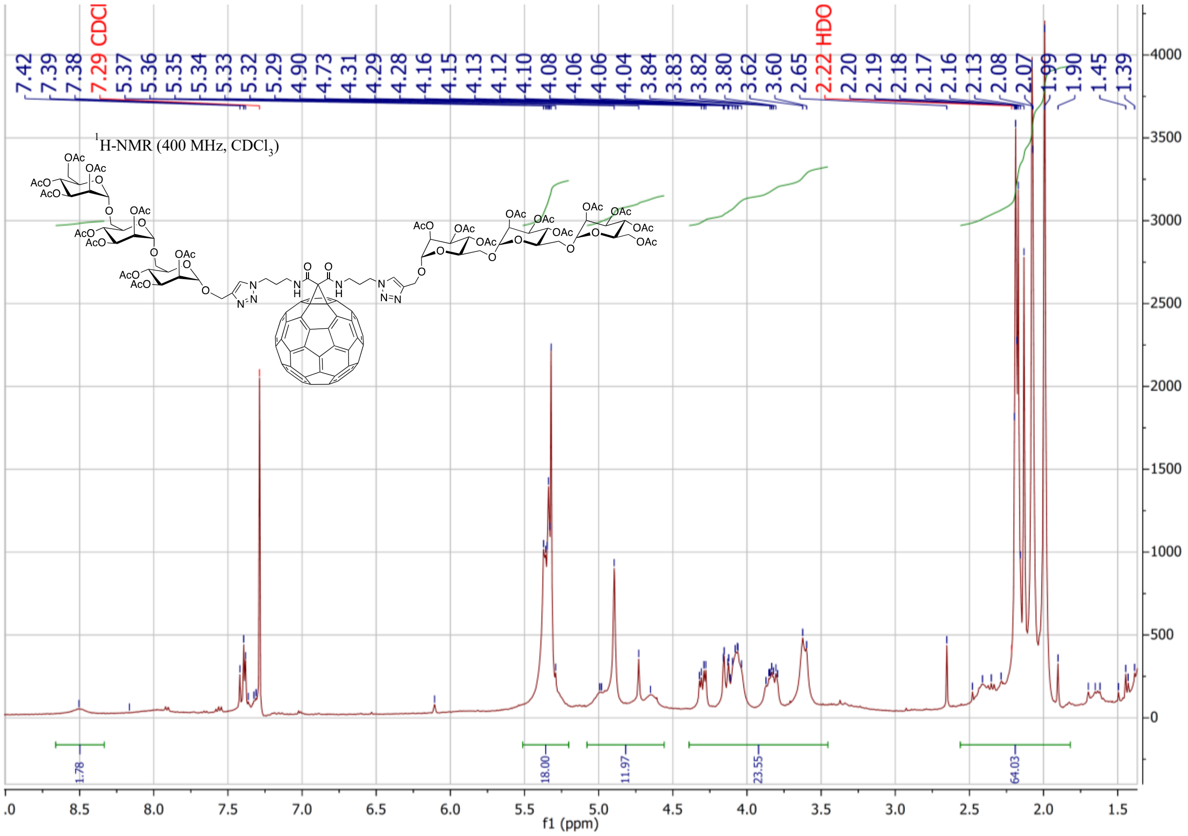
7.42
7.39
7.38
7.29 CDCl
5.37
5.36
5.35
5.34
5.33
5.32
5.29
4.90
4.73
4.31
4.29
4.28
4.16
4.15
4.13
4.12
4.10
4.08
4.06
4.06
4.04
3.84
3.83
3.82
3.80
3.62
3.60
2.65
2.22 HDO
2.20
2.19
2.18
2.17
2.16
2.13
2.08
2.07
1.99
1.90
1.45
1.39
1H-NMR (400 MHz, CDCl3)
1.78
18.00
11.97
23.55
64.03
f1 (ppm)

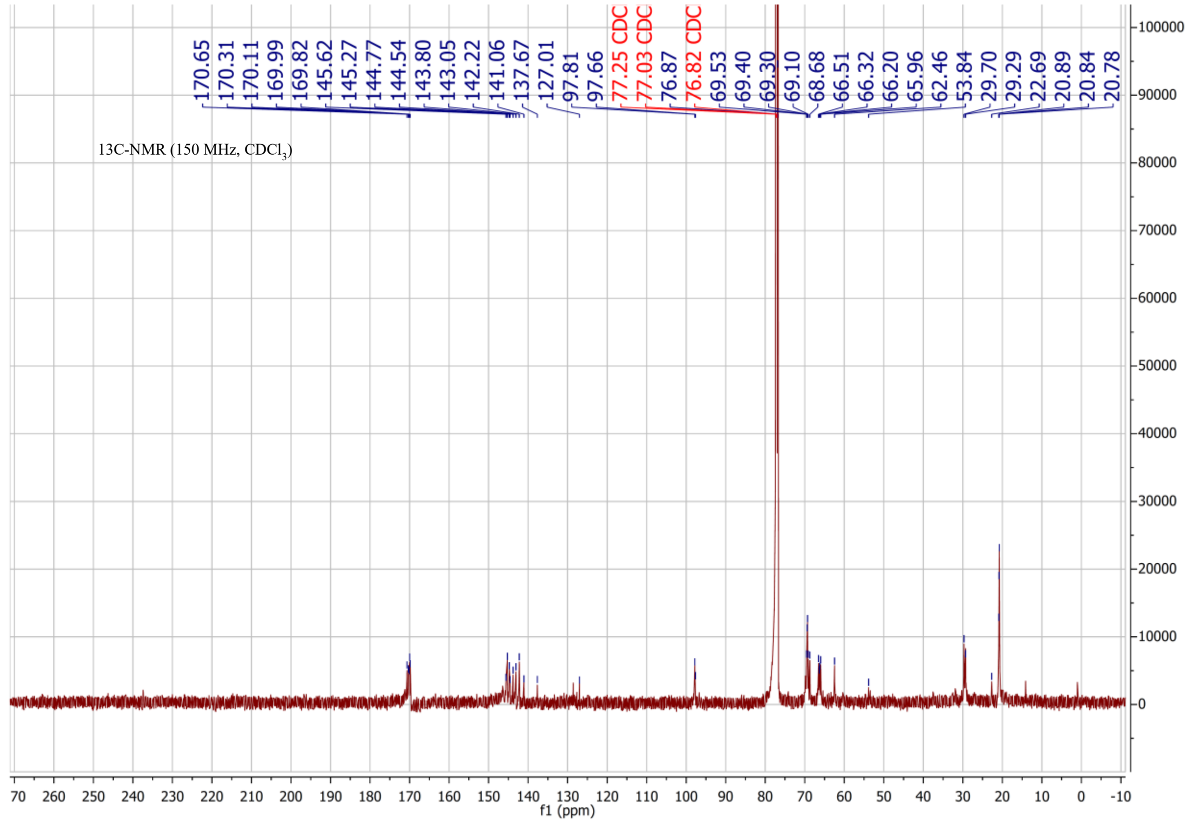
170.65
170.31
170.11
169.99
169.82
145.62
145.27
144.77
144.54
143.80
143.05
142.22
141.06
137.67
127.01
97.81
97.66
77.25 CDC
77.03 CDC
76.87
76.82 CDC
69.53
69.40
69.30
69.10
68.68
66.51
66.32
66.20
65.96
62.46
53.84
29.70
29.29
22.69
20.89
20.84
20.78
13C-NMR (150 MHz, CDCl3)
f1 (ppm)

Display Report

Analysis Info

Analysis Name	D:\Data\GOBS\Lisa\LT C60 TRIS 1,6 1,6 HRMS .d	Acquisition Date	11/25/2020 12:01:23 PM
Method	tune_high.m	Operator	BDAL@DE
Sample Name	LT C60 TRIS 1,6 1,6 HRMS	Instrument / Ser#	micrOTOF 235
Comment			

Acquisition Parameter

Source Type	ESI	Ion Polarity	Positive	Set Nebulizer	5.8 psi
Focus	Active			Set Dry Heater	180 °C
Scan Begin	50 m/z	Set Capillary	4500 V	Set Dry Gas	4.0 l/min
Scan End	4000 m/z	Set End Plate Offset	-500 V	Set Divert Valve	Source

Meas. m/z	z	#	Formula	m/z	err [ppm]	mSigma	err [mDa]
2934.6875	1+	1	C 151 H 122 N 8 Na O 54	2933.6939	1.9	34.9	5.6

Meas. m/z	z	#	Formula	m/z	err [ppm]	mSigma	err [mDa]

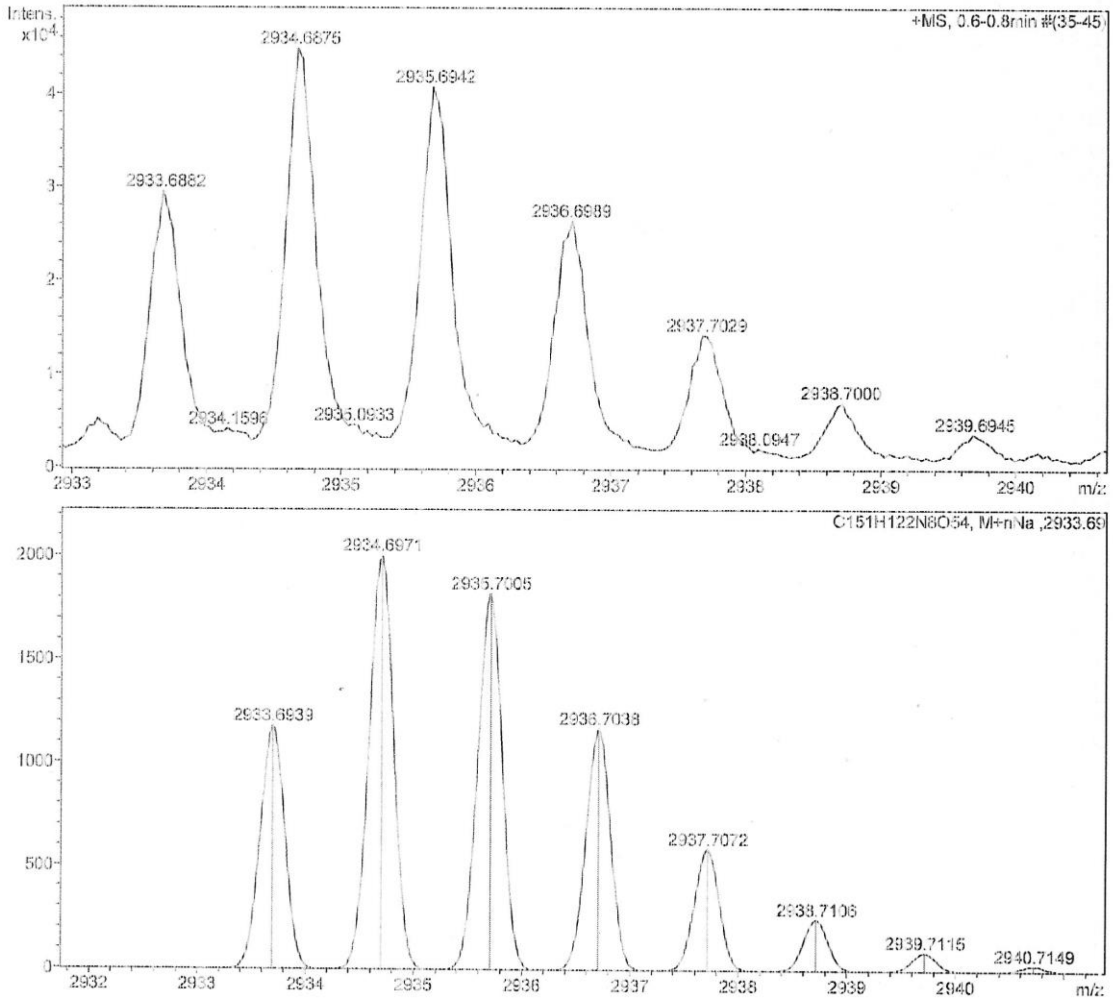

Compound 18

^{1}H-NMR (400 MHz, $CDCl_3$)

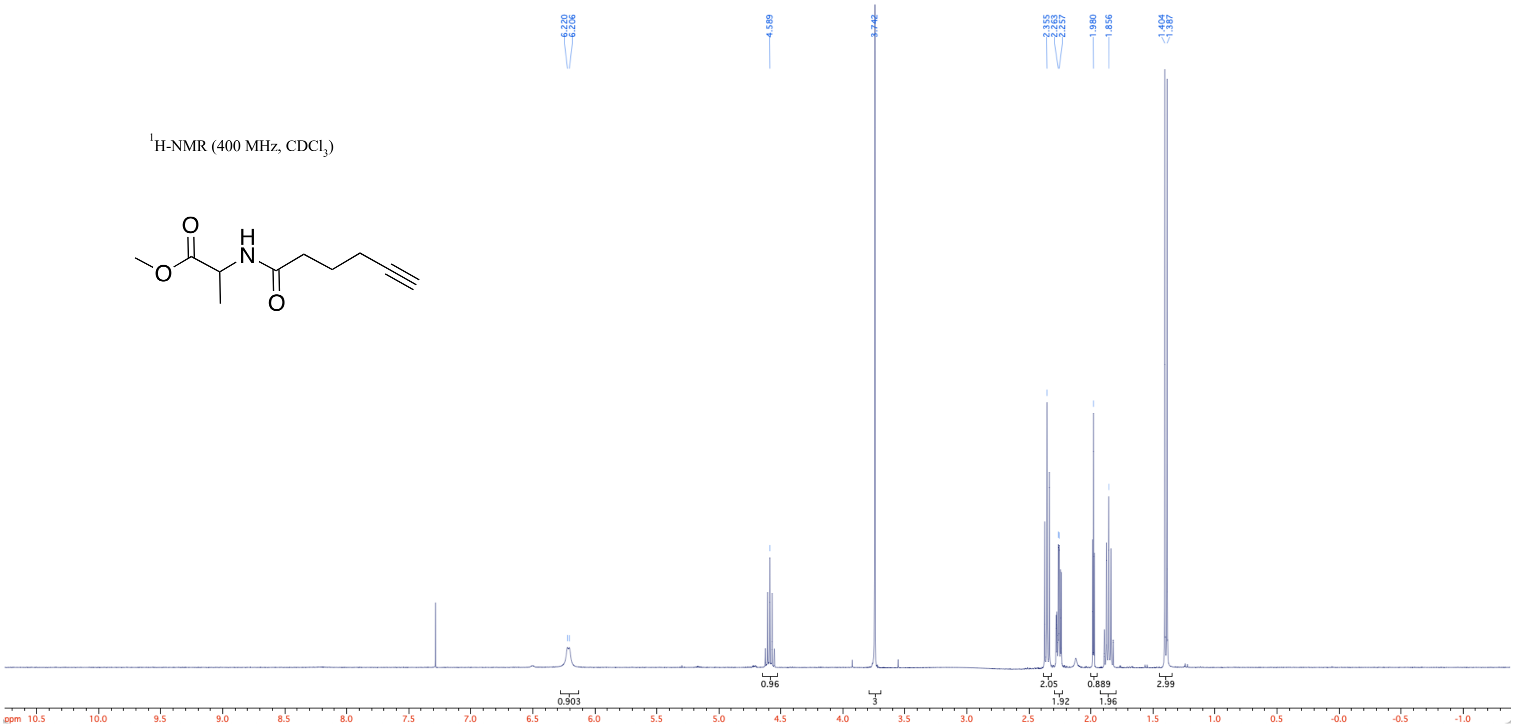

13C-NMR (100 MHz, $CDCl_3$)

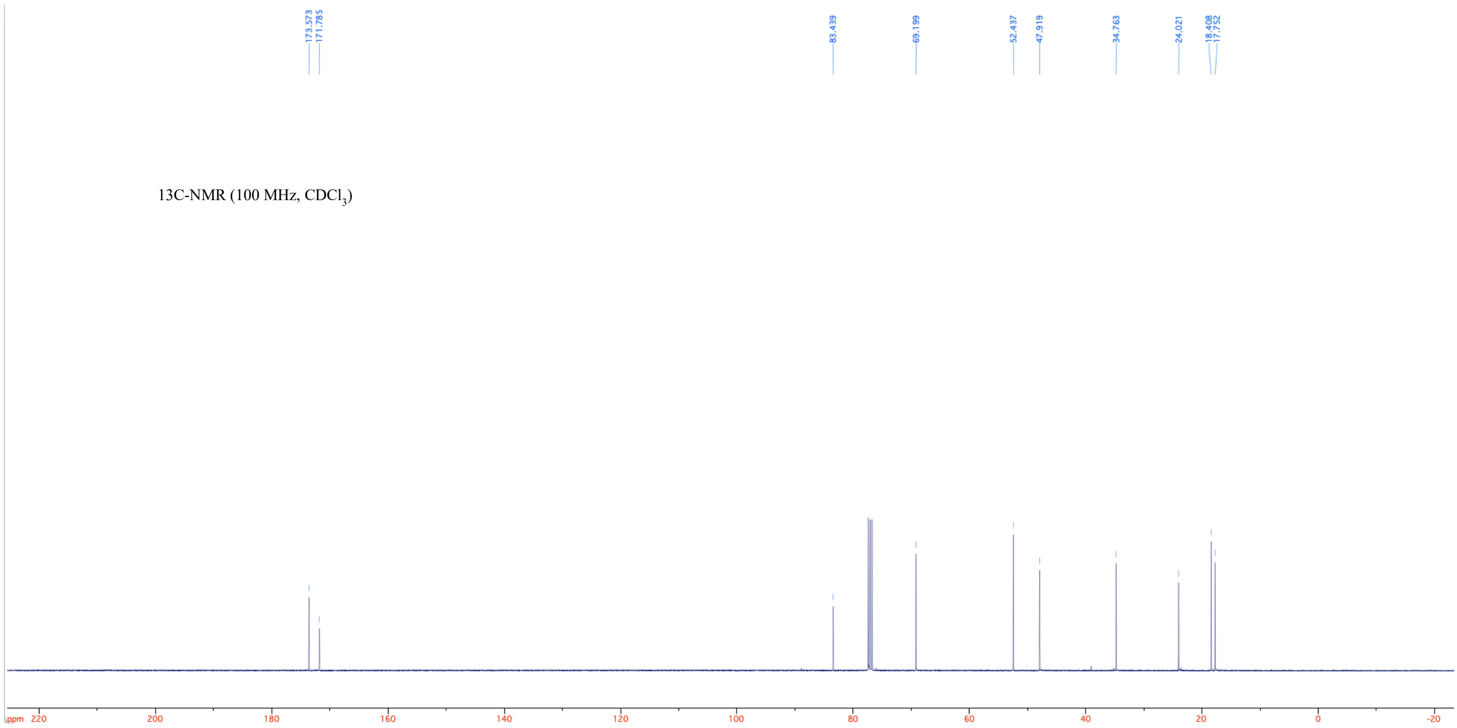

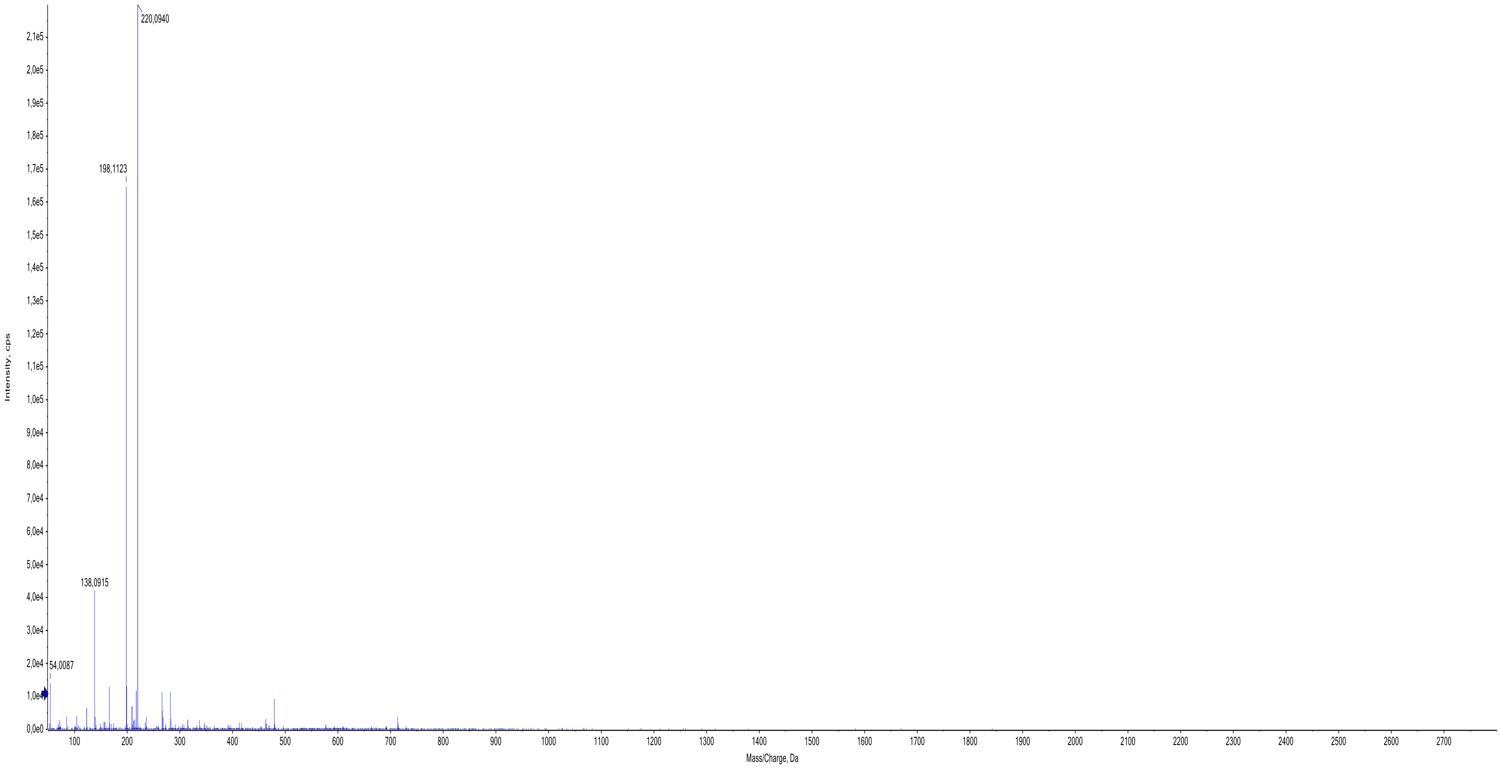

220,0940
198,1123
138,0915
54,0087
Intensity, cps
Mass/Charge, Da

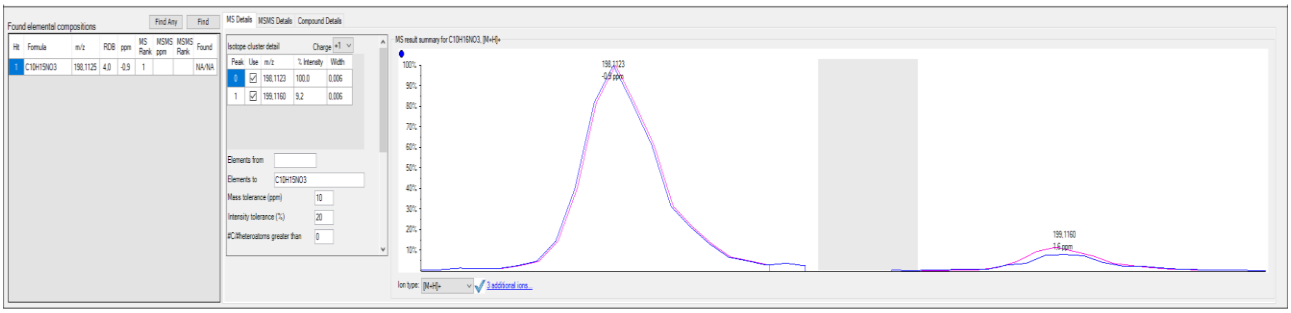

Found elemental compositions
Find Any
Find
Hit Formula m/z RDB ppm MS Rank MSMS ppm MSMS Rank Found
1 C10H15NO3 198,1125 4,0 -0,9 1 NA/NA
MS Details MSMS Details Compound Details
Isotope cluster detail
Charge +1
Peak Use m/z % Intensity Width
0 198,1123 100,0 0,006
1 199,1160 9,2 0,006
Elements from
Elements to C10H15NO3
Mass tolerance (ppm) 10
Intensity tolerance (%) 20
#C/#heteroatoms greater than 0
MS result summary for C10H16NO3, [M+H]+
198,1123
-0,9 ppm
199,1160
1,6 ppm
Ion type: [M+H]+
3 additional ions...

Compound 19

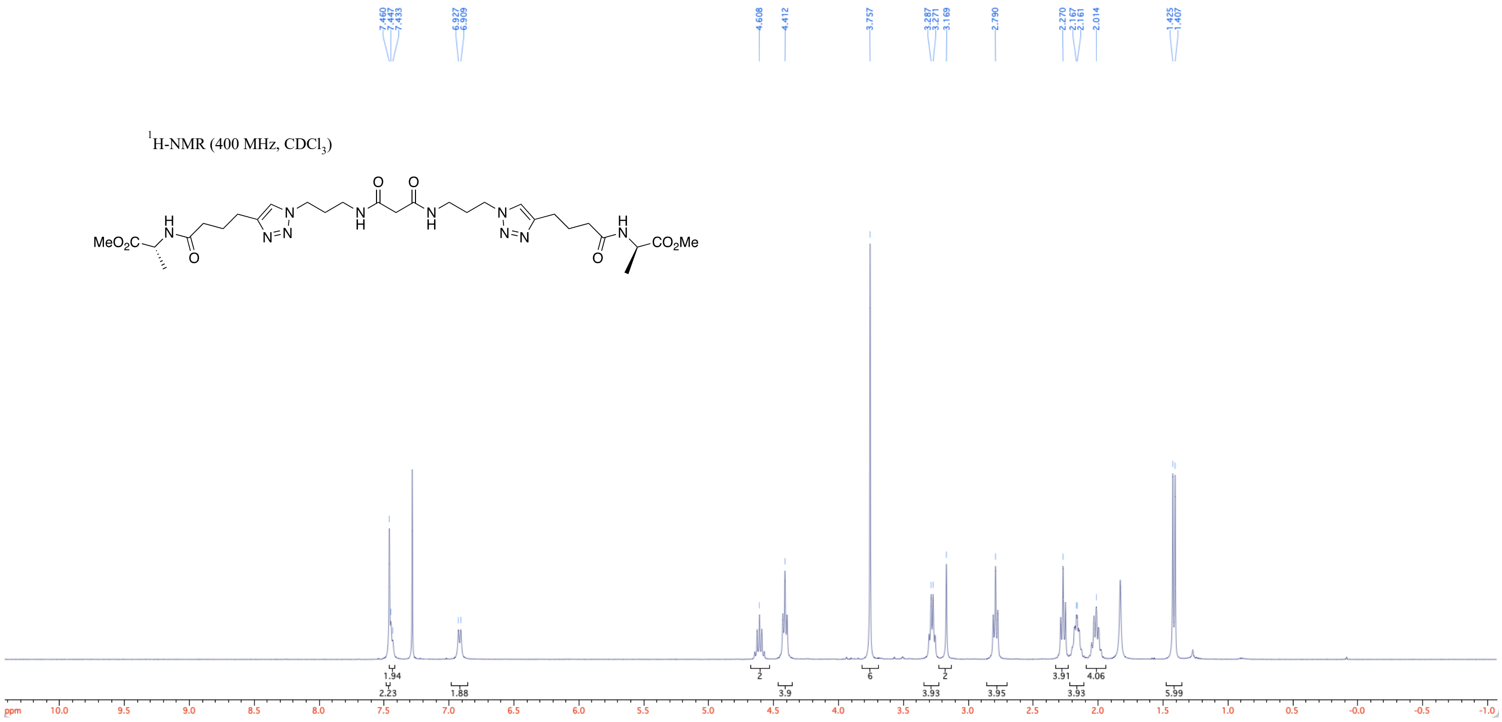

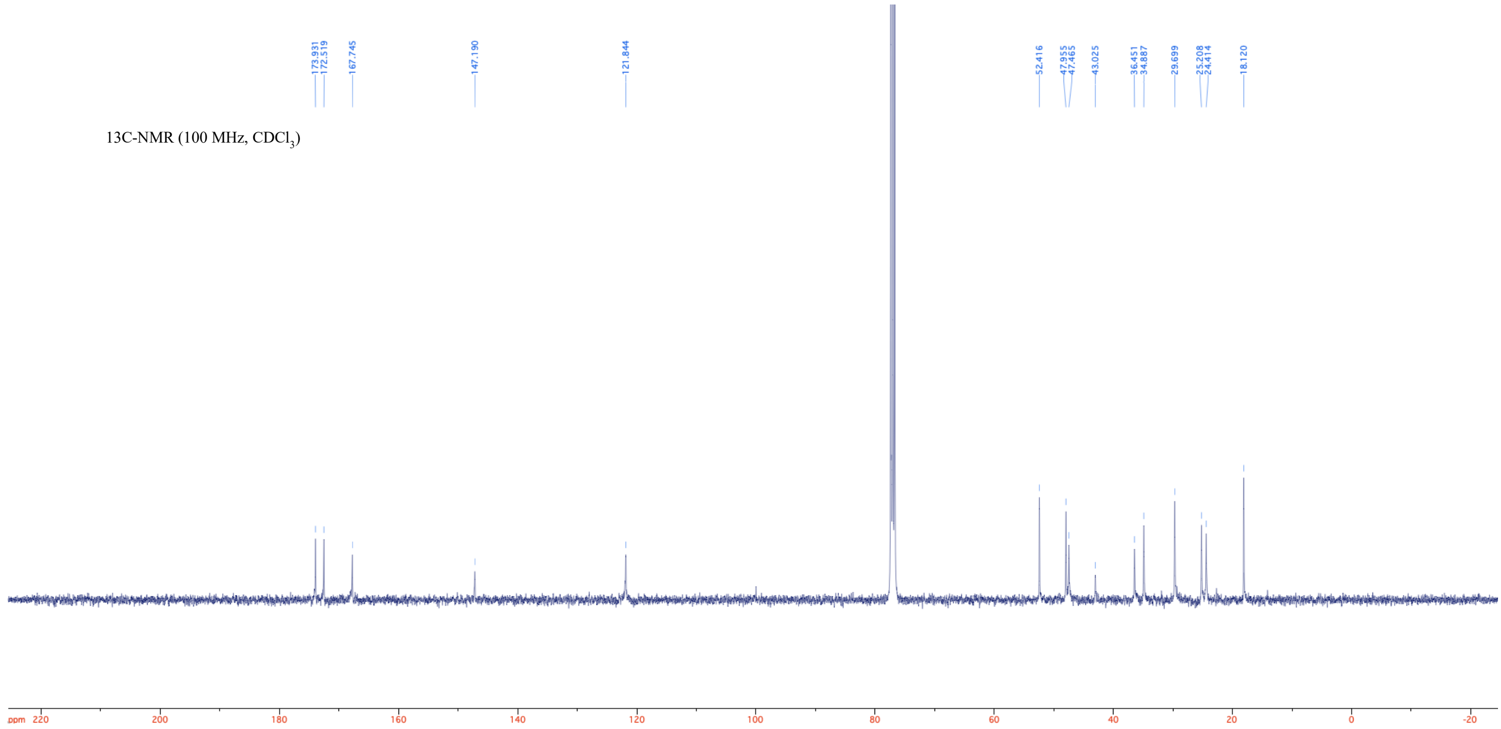

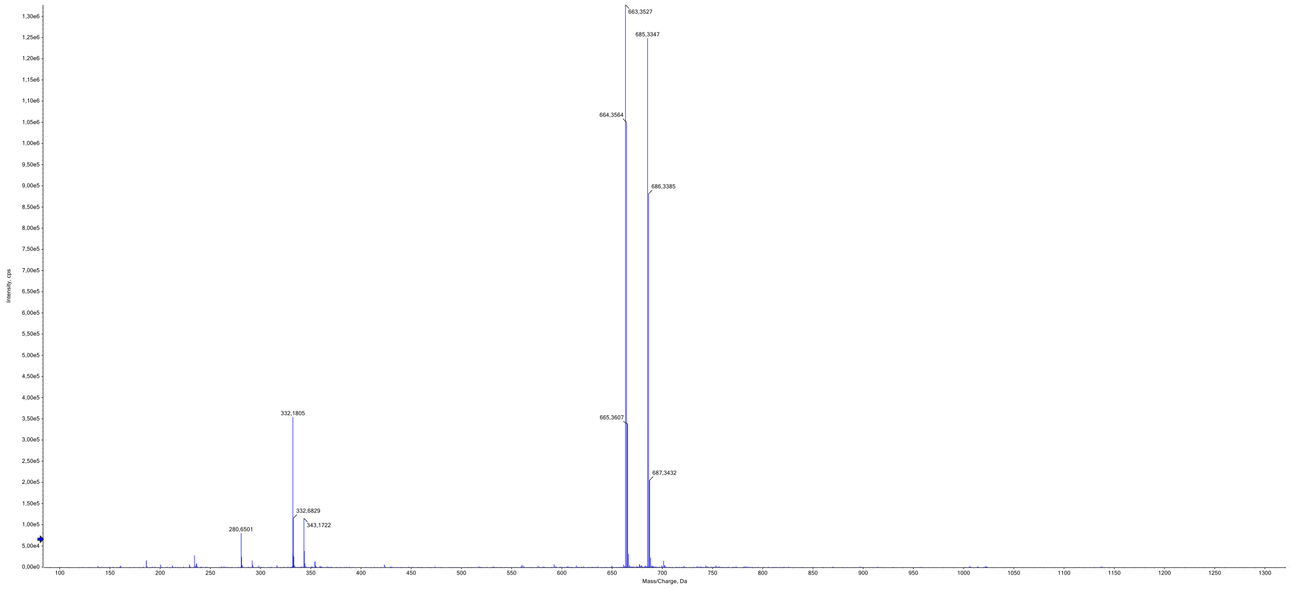
663,3527
685,3347
664,3564
686,3385
332,1805
665,3607
687,3432
332,6829
343,1722
280,6501
Intensity, cps
Mass/Charge, Da

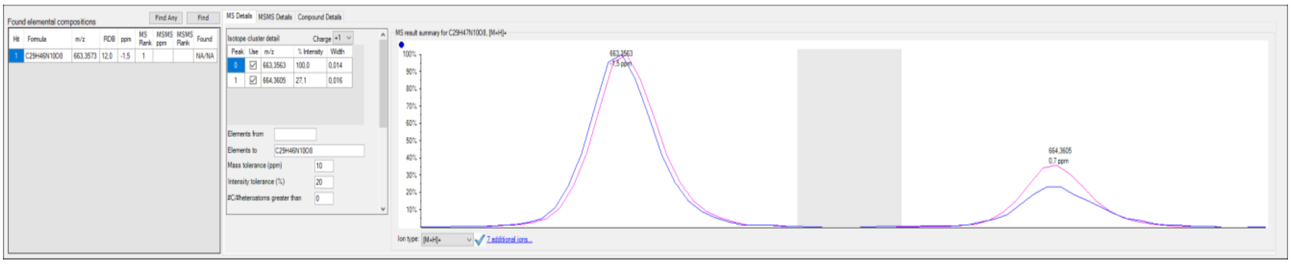
Found elemental compositions
Find Any
Find
Hit | Formula | m/z | RDB | ppm | MS Rank | MSMS ppm | MSMS Rank | Found
1 | C29H46N10O8 | 663,3573 | 12,0 | -1,5 | 1 | | | NA/NA
MS Details
MSMS Details
Compound Details
Isotope cluster detail
Charge +1
Peak | Use | m/z | % Intensity | Width
0 | 663,3563 | 100,0 | 0,014
1 | 664,3605 | 27,1 | 0,016
Elements from
Elements to C29H46N10O8
Mass tolerance (ppm) 10
Intensity tolerance (%) 20
#C/#heteroatoms greater than 0
MS result summary for C29H47N10O8, [M+H]+
663,3563
-1,5 ppm
664,3605
0,7 ppm
Ion type: [M+H]+
7 additional ions...

Compound 20

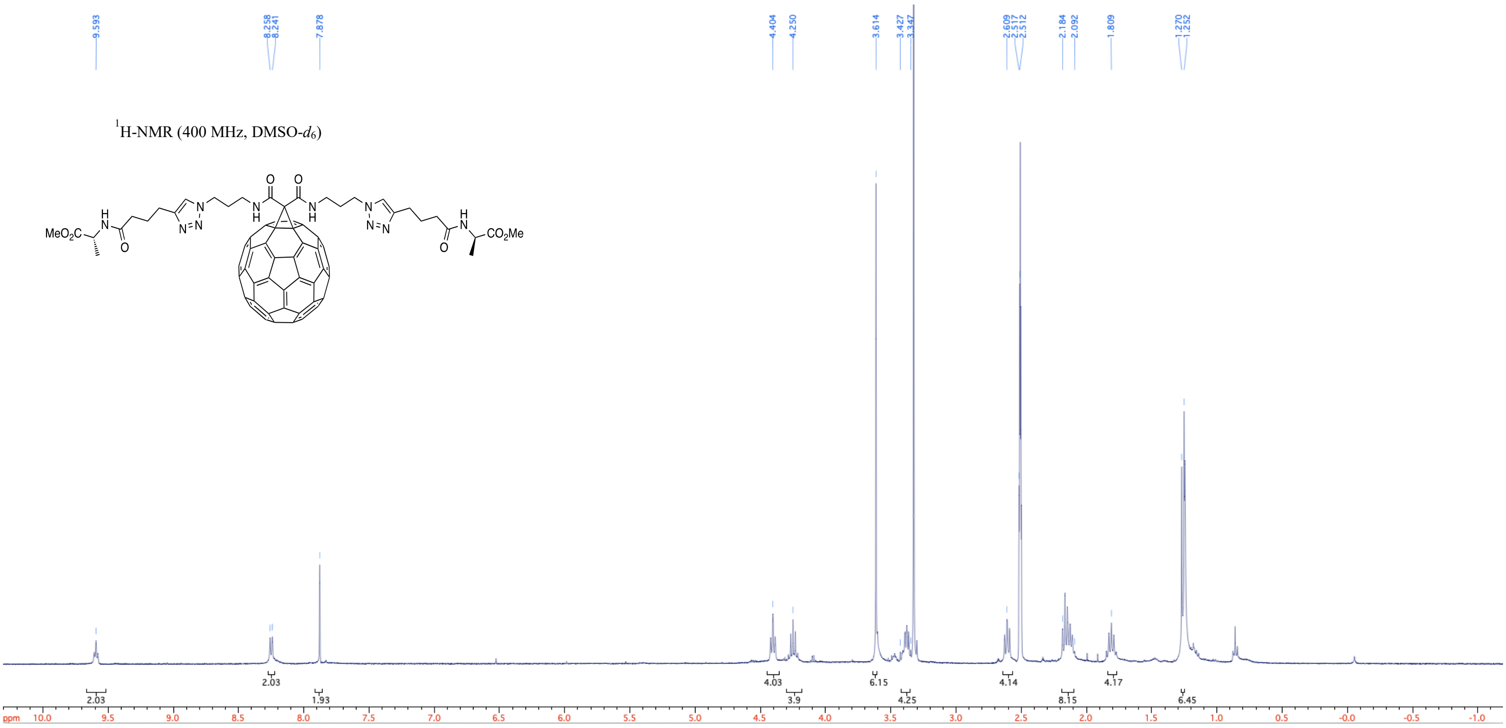

9.593
8.258
8.241
7.878
4.404
4.250
3.614
3.427
3.347
2.609
2.517
2.512
2.184
2.092
1.809
1.270
1.252
¹H-NMR (400 MHz, DMSO-d6)
MeO2C
CO2Me
2.03
2.03
1.93
4.03
3.9
6.15
4.25
4.14
8.15
4.17
6.45
ppm

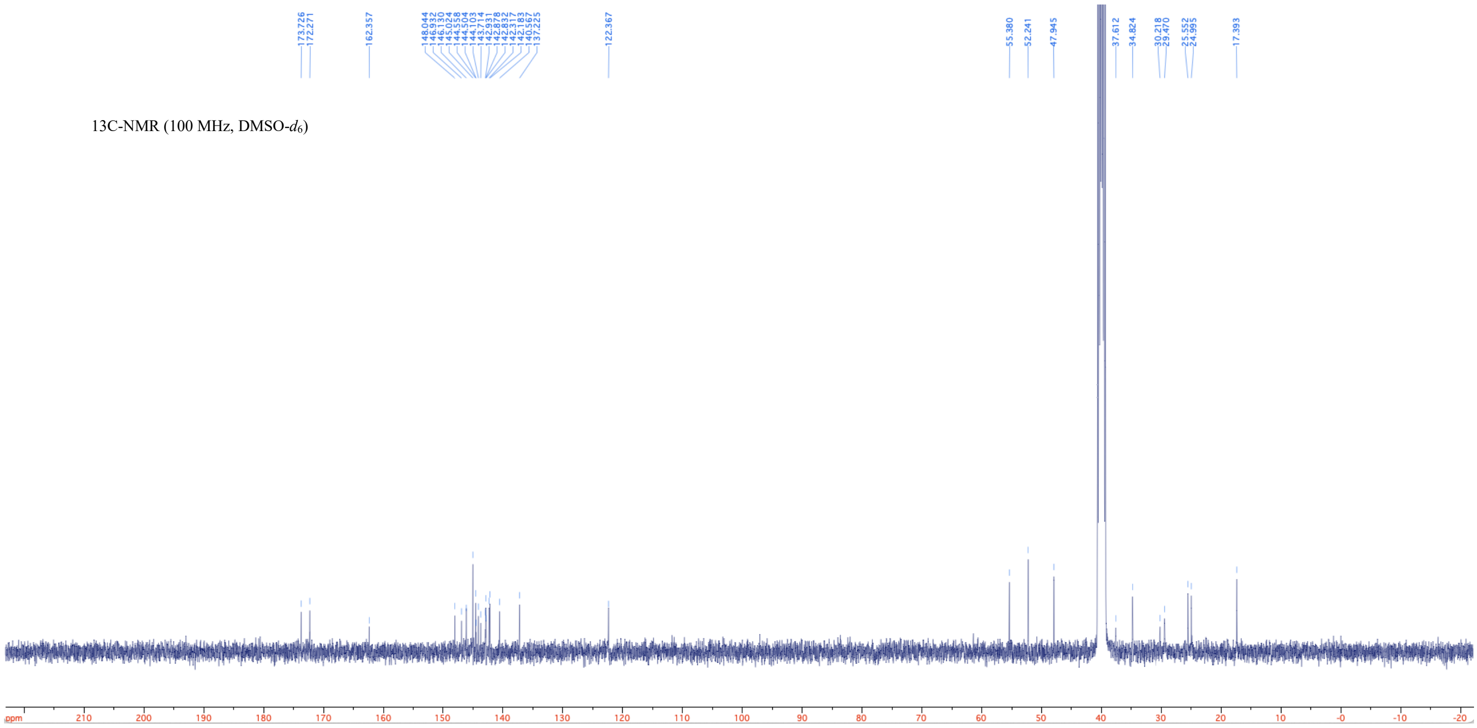

173.726
172.271
162.357
148.044
146.932
146.130
145.024
144.558
144.504
144.103
143.714
142.931
142.878
142.832
142.317
141.183
140.567
137.225
122.367
55.380
52.241
47.945
37.612
34.824
30.218
29.470
25.552
24.995
17.393
13C-NMR (100 MHz, DMSO-d6)
ppm

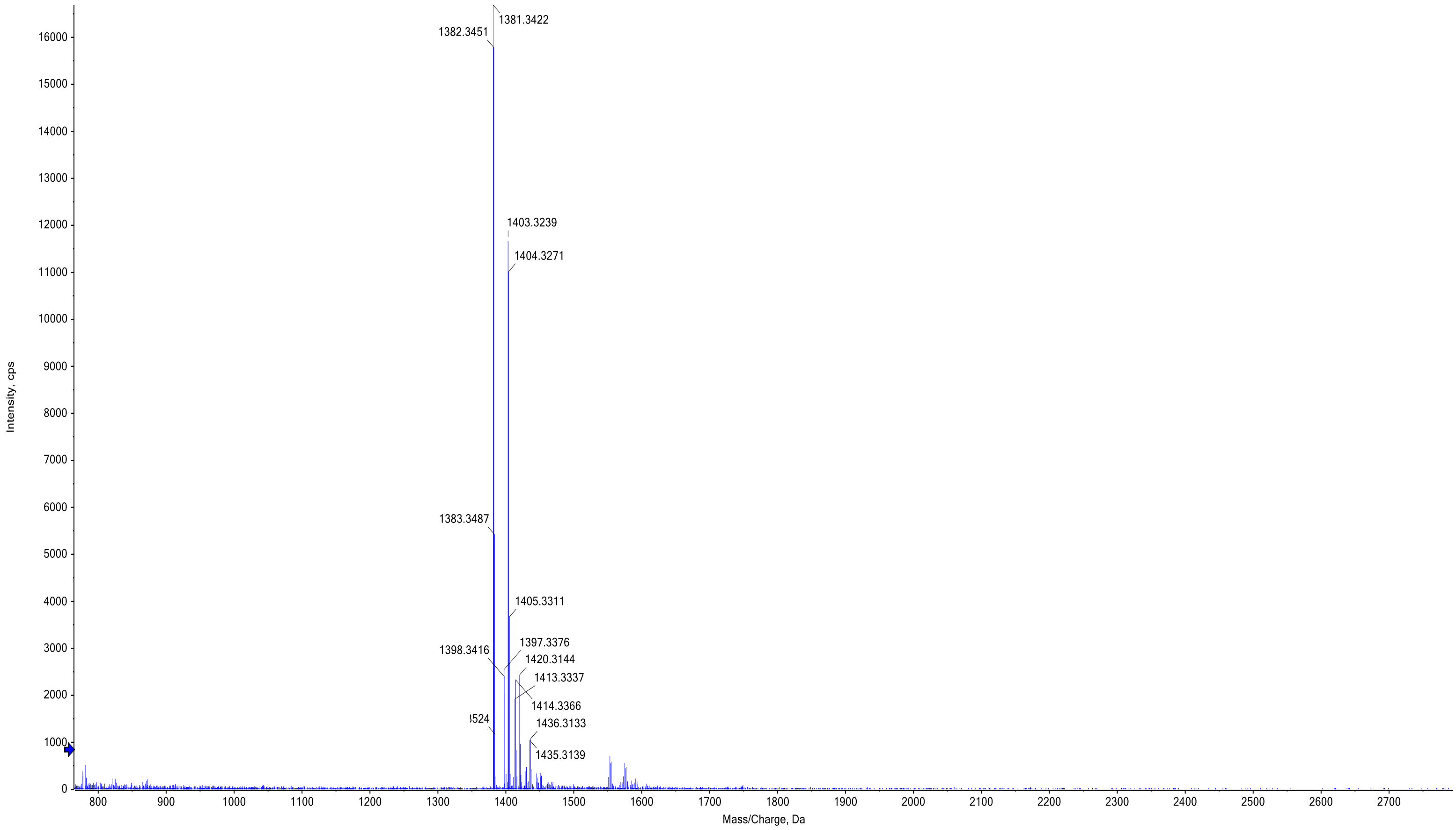
1381.3422
1382.3451
1403.3239
1404.3271
1383.3487
1405.3311
1398.3416
1397.3376
1420.3144
1413.3337
1414.3366
1436.3133
1435.3139
Intensity, cps
Mass/Charge, Da

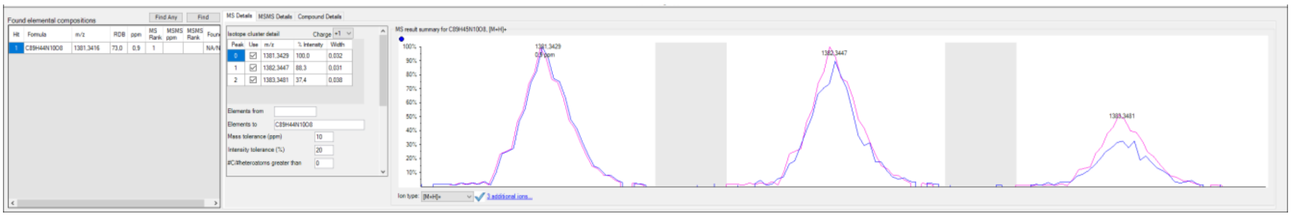

Compound 21

^{1}H-NMR (400 MHz, $CDCl_3$)

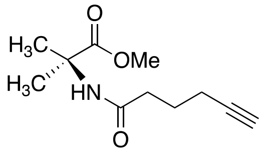

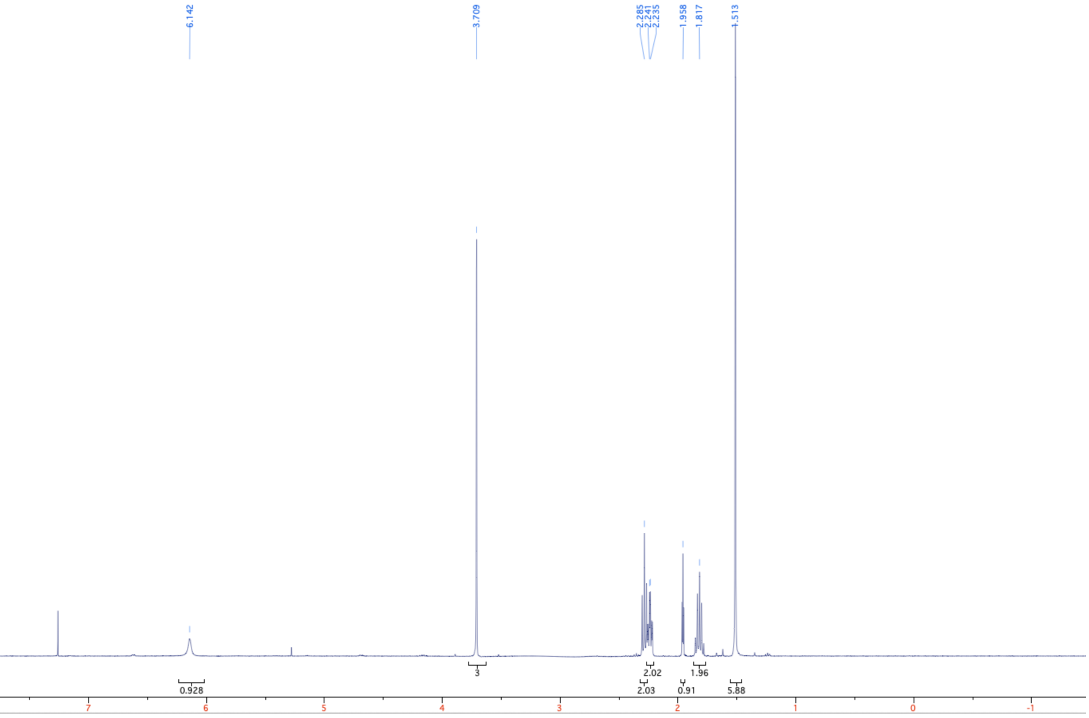

13C-NMR (100 MHz, $CDCl_3$)

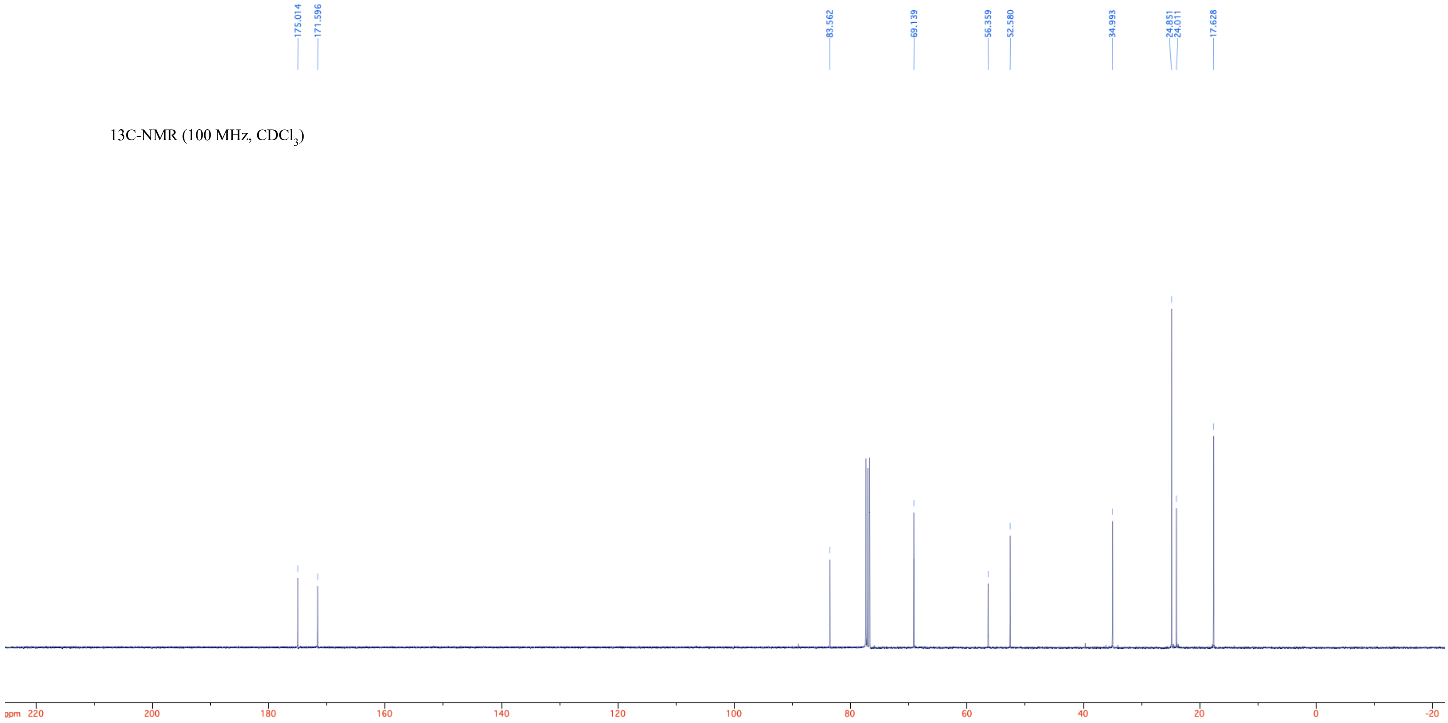

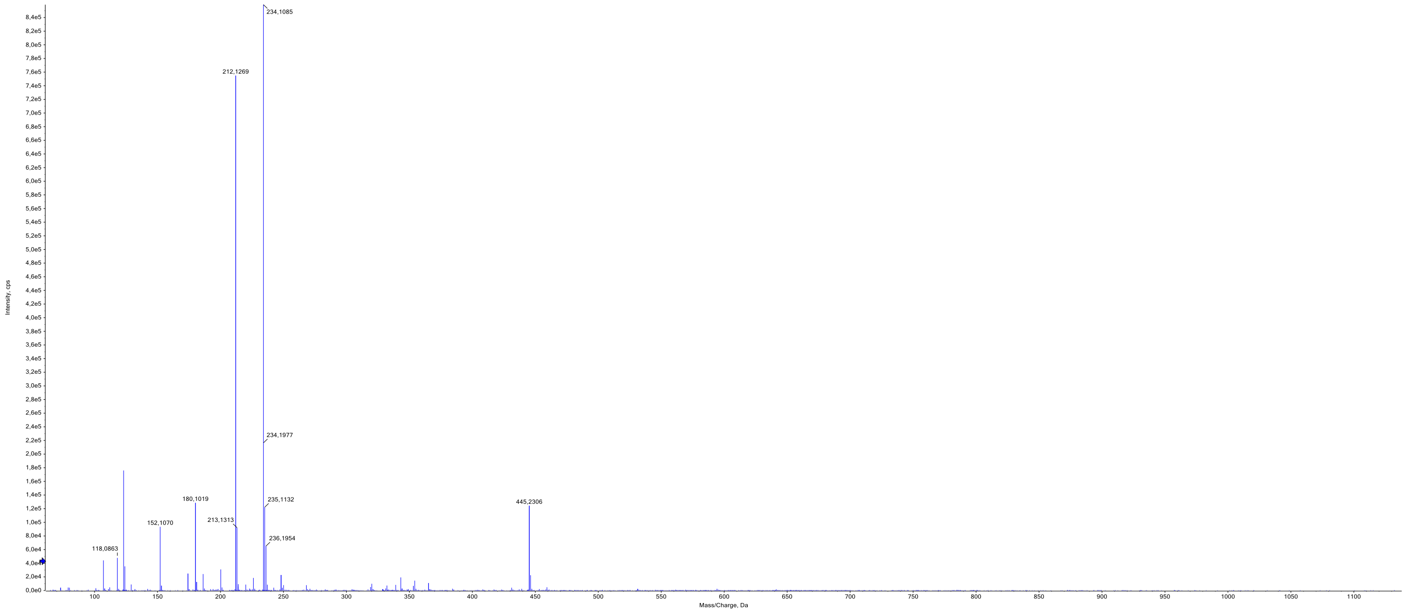
234,1085
212,1269
234,1977
180,1019
235,1132
445,2306
213,1313
152,1070
236,1954
118,0863
Intensity, cps
Mass/Charge, Da

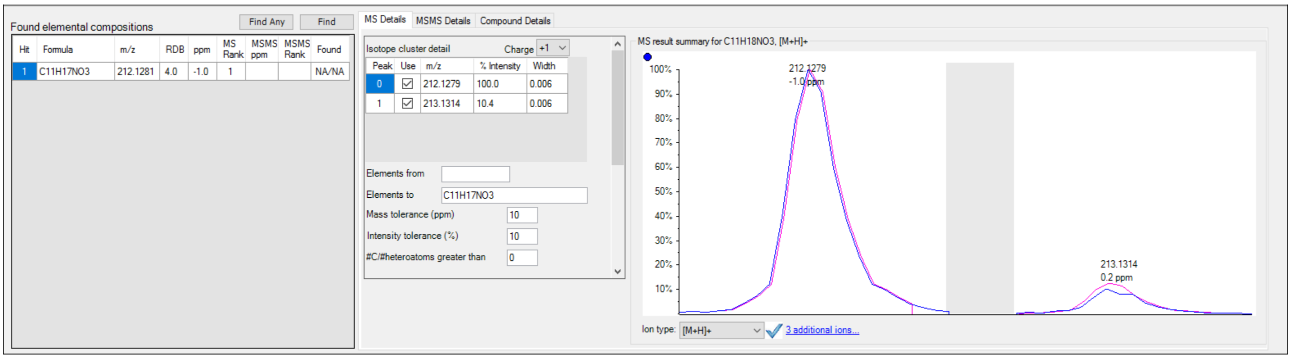
Found elemental compositions
Find Any
Find
Hit | Formula | m/z | RDB | ppm | MS Rank | MSMS ppm | MSMS Rank | Found
1 | C11H17NO3 | 212.1281 | 4.0 | -1.0 | 1 | | | NA/NA
MS Details
MSMS Details
Compound Details
Isotope cluster detail
Charge +1
Peak | Use | m/z | % Intensity | Width
0 | 212.1279 | 100.0 | 0.006
1 | 213.1314 | 10.4 | 0.006
Elements from
Elements to C11H17NO3
Mass tolerance (ppm) 10
Intensity tolerance (%) 10
#C/#heteroatoms greater than 0
MS result summary for C11H18NO3, [M+H]+
212.1279
-1.0 ppm
213.1314
0.2 ppm
Ion type: [M+H]+
3 additional ions...

Compound 22

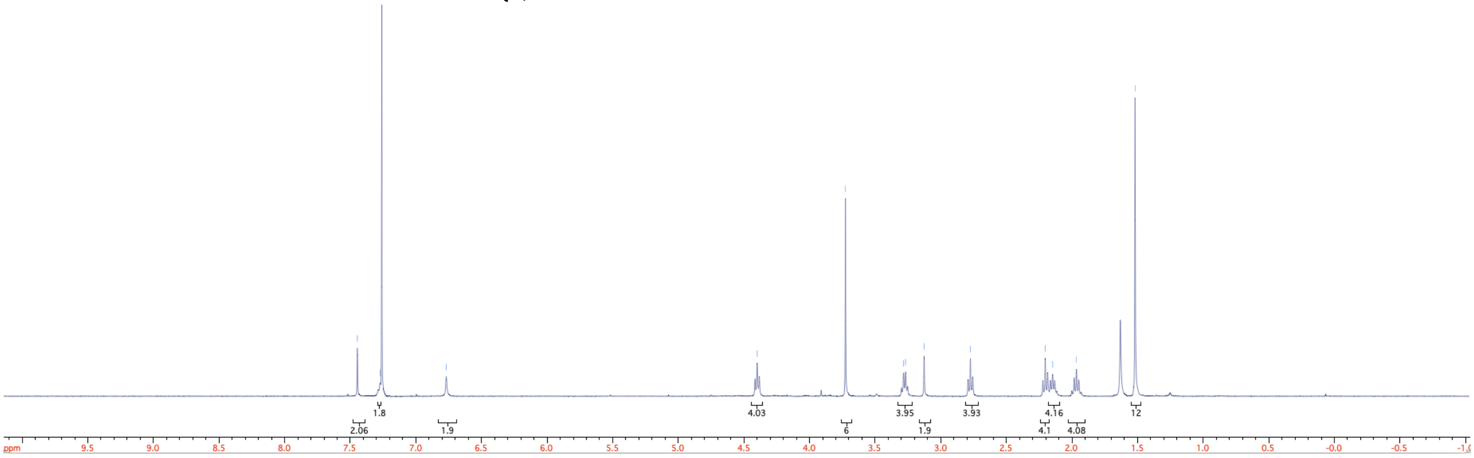

^{1}H-NMR (400 MHz, $CDCl_3$)

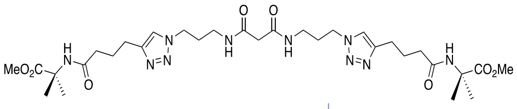

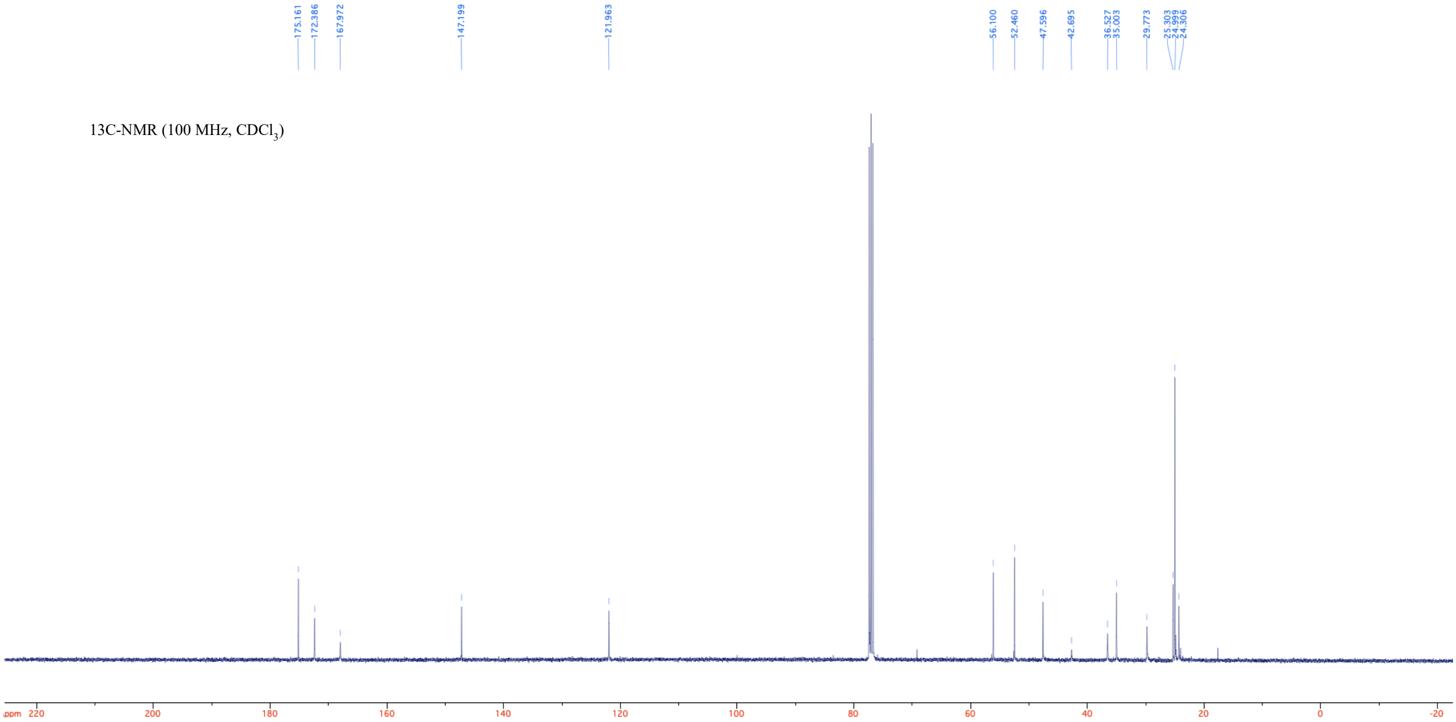

13C-NMR (100 MHz, $CDCl_3$)

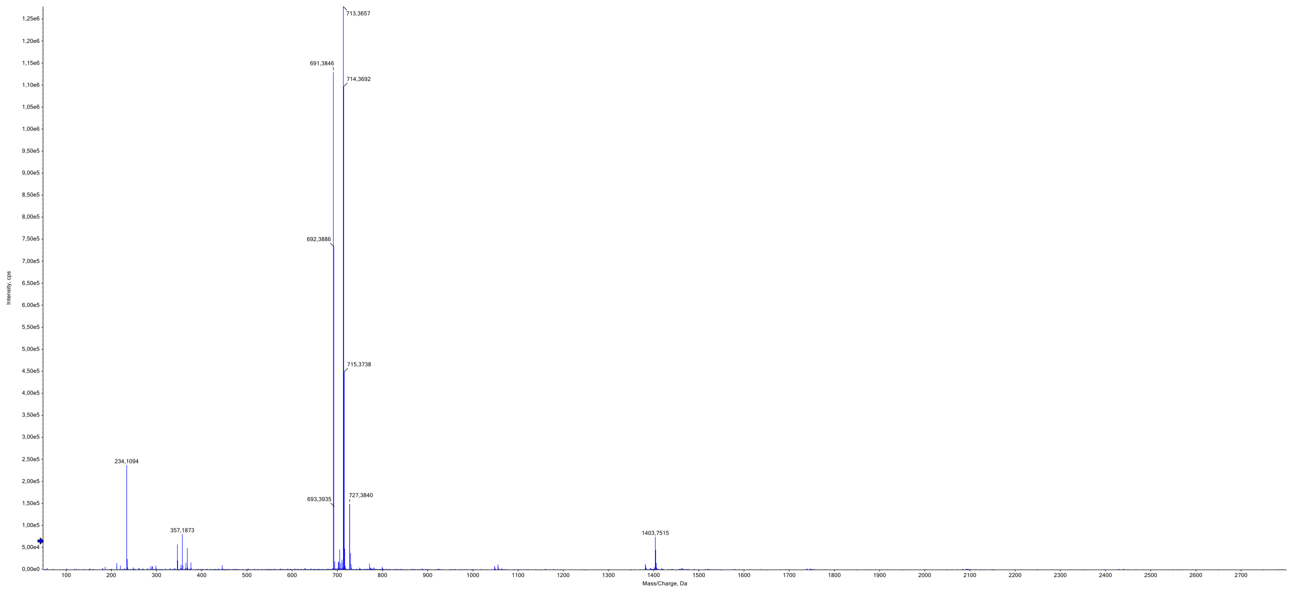
713,3657
691,3846
714,3692
692,3886
715,3738
234,1094
693,3935
727,3840
357,1873
1403,7515
Intensity, cps
Mass/Charge, Da

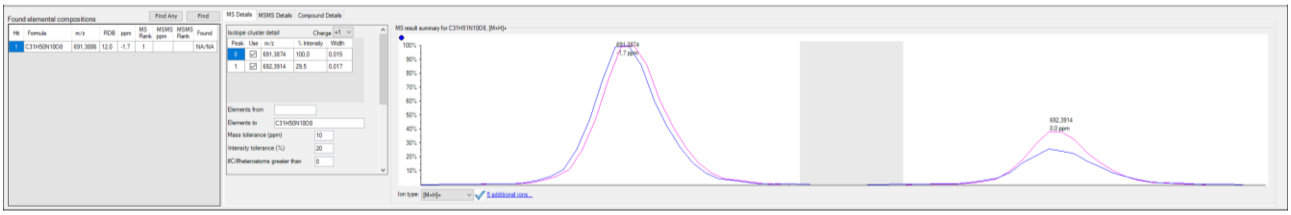
Found elemental compositions
Find Any
Find
Hit | Formula | m/z | RDB | ppm | MS Rank | MSMS ppm | MSMS Rank | Found
1 | C31H50N10O8 | 691,3886 | 12,0 | -1,7 | 1 | | | NA/NA
MS Details
MSMS Details
Compound Details
Isotope cluster detail
Charge +1
Peak | Use | m/z | % Intensity | Width
0 | 691,3874 | 100,0 | 0,015
1 | 692,3914 | 29,5 | 0,017
Elements from
Elements to C31H50N10O8
Mass tolerance (ppm) 10
Intensity tolerance (%) 20
#C/#heteroatoms greater than 0
MS result summary for C31H51N10O8, [M+H]+
691,3874
-1,7 ppm
692,3914
0,0 ppm
Ion type: [M+H]+
5 additional ions...

Compound 23

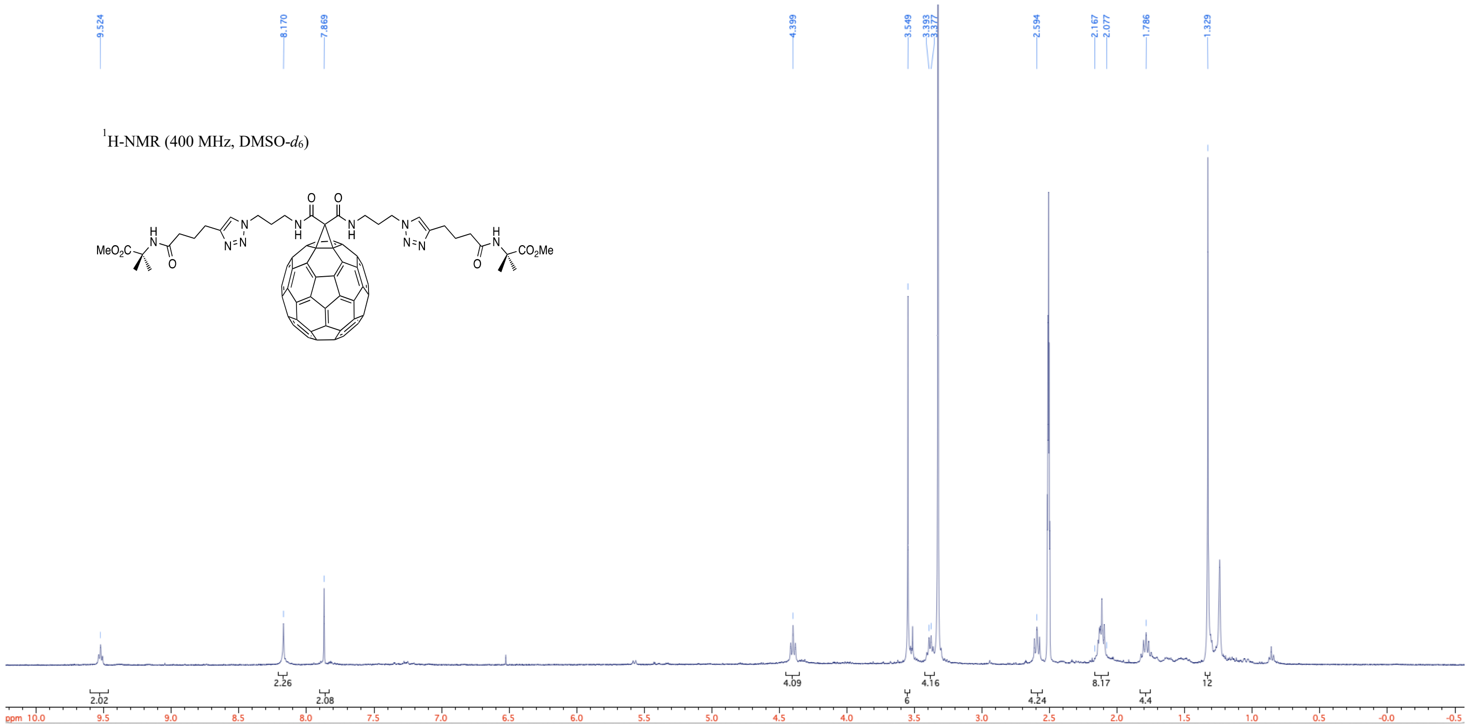

9.524
8.170
7.869
4.399
3.549
3.393
3.377
2.594
2.167
2.077
1.786
1.329
1H-NMR (400 MHz, DMSO-d6)
MeO2C
CO2Me
2.02
2.26
2.08
4.09
6
4.16
4.24
8.17
4.4
12
ppm

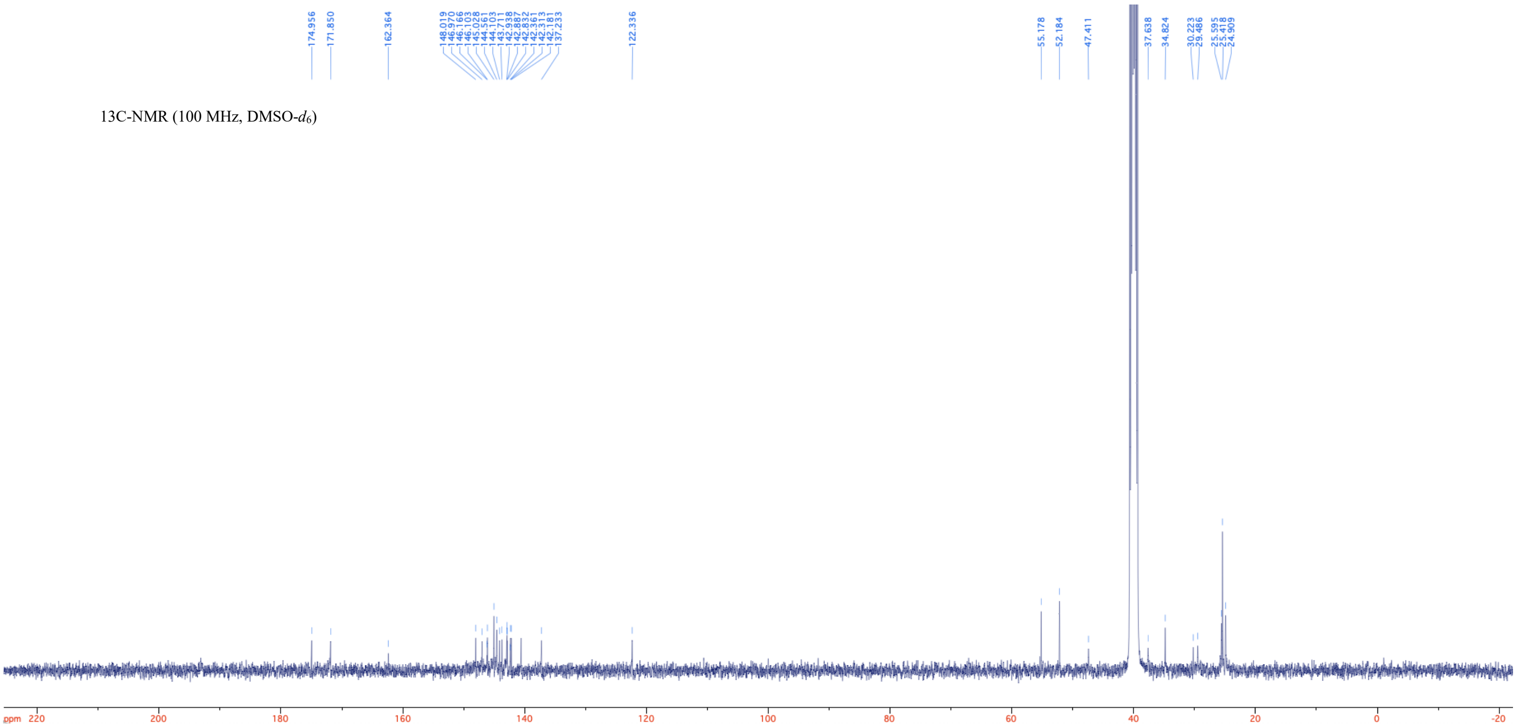

174.956
171.850
162.364
148.019
146.970
146.166
146.103
145.028
144.551
144.103
143.711
142.938
142.887
142.832
142.361
142.313
142.181
137.233
122.336
55.178
52.184
47.411
37.638
34.824
30.223
29.486
25.595
25.418
24.909
13C-NMR (100 MHz, DMSO-d6)
ppm

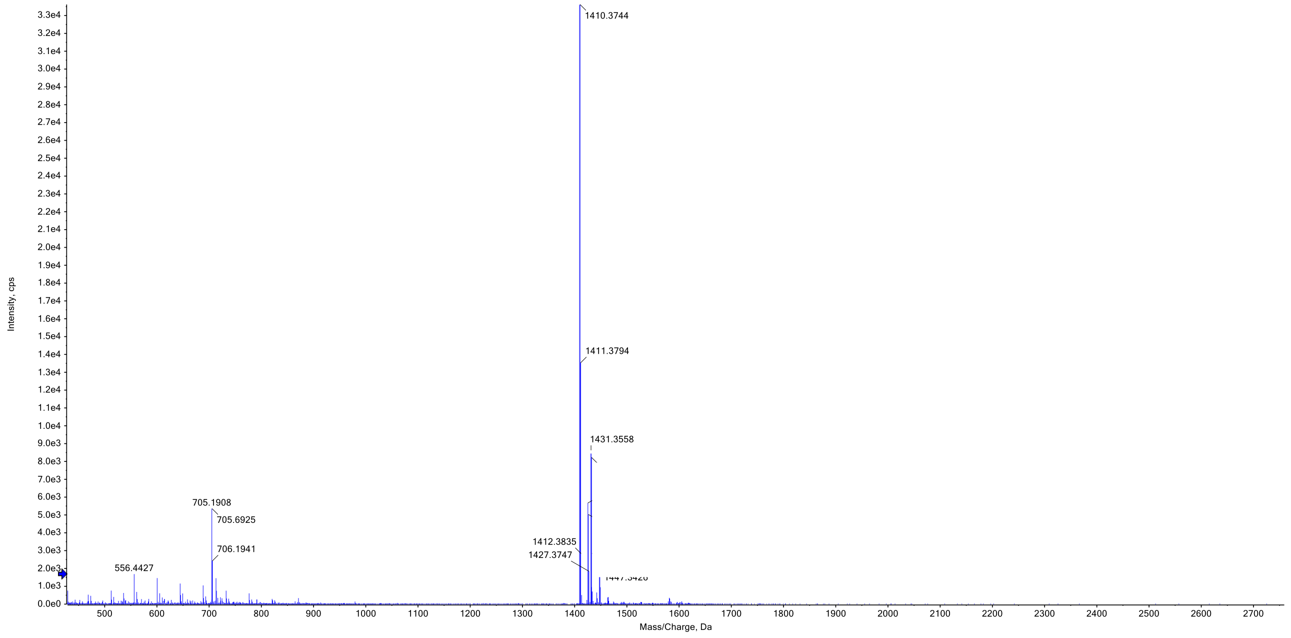
1410.3744
1411.3794
1431.3558
705.1908
705.6925
706.1941
556.4427
1412.3835
1427.3747
Intensity, cps
Mass/Charge, Da

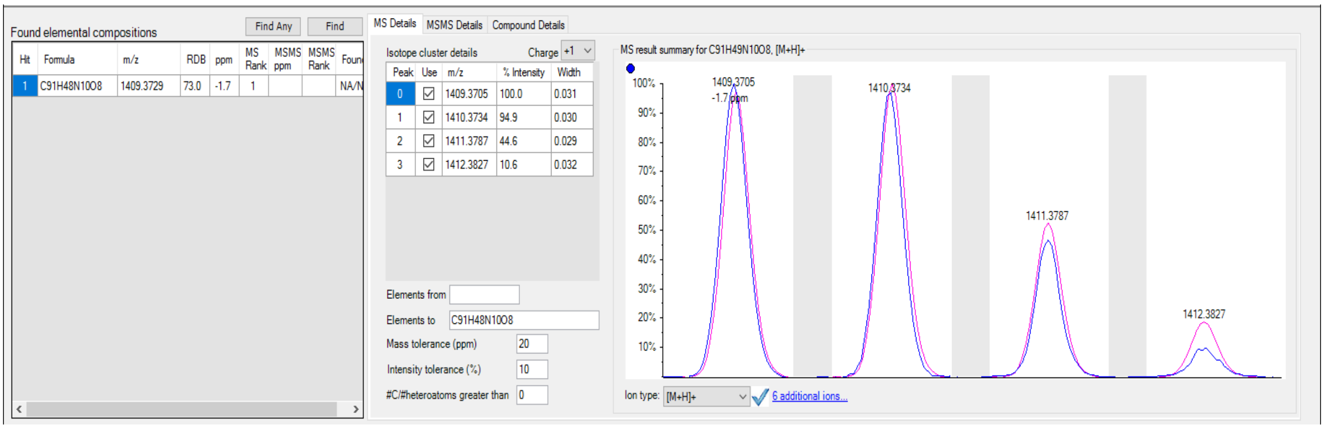
Found elemental compositions
Find Any
Find
Hit | Formula | m/z | RDB | ppm | MS Rank | MSMS ppm | MSMS Rank
1 | C91H48N10O8 | 1409.3729 | 73.0 | -1.7 | 1
MS Details
MSMS Details
Compound Details
Isotope cluster details
Charge +1
Peak | Use | m/z | % Intensity | Width
0 | 1409.3705 | 100.0 | 0.031
1 | 1410.3734 | 94.9 | 0.030
2 | 1411.3787 | 44.6 | 0.029
3 | 1412.3827 | 10.6 | 0.032
Elements from
Elements to C91H48N10O8
Mass tolerance (ppm) 20
Intensity tolerance (%) 10
#C/#heteroatoms greater than 0
MS result summary for C91H49N10O8, [M+H]+
1409.3705
-1.7 ppm
1410.3734
1411.3787
1412.3827
Ion type: [M+H]+
6 additional ions...

Compound 24

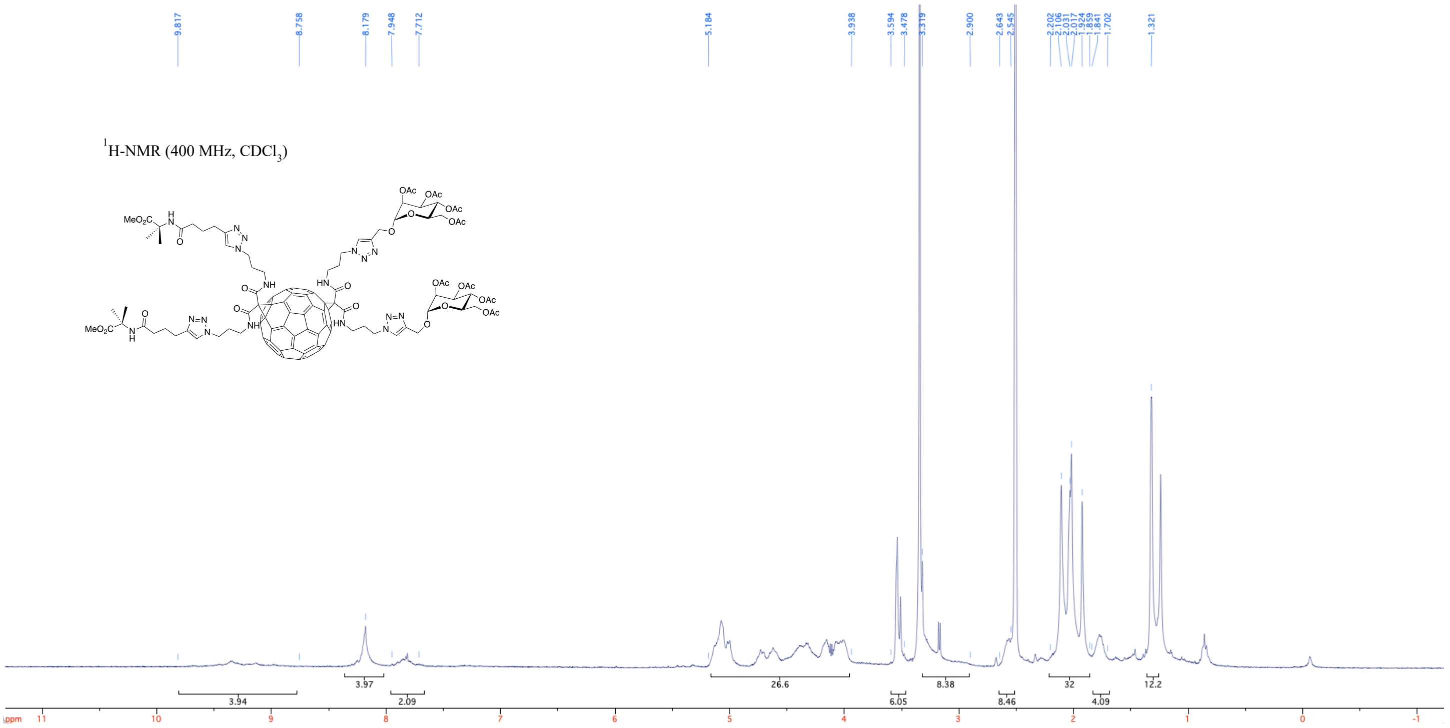

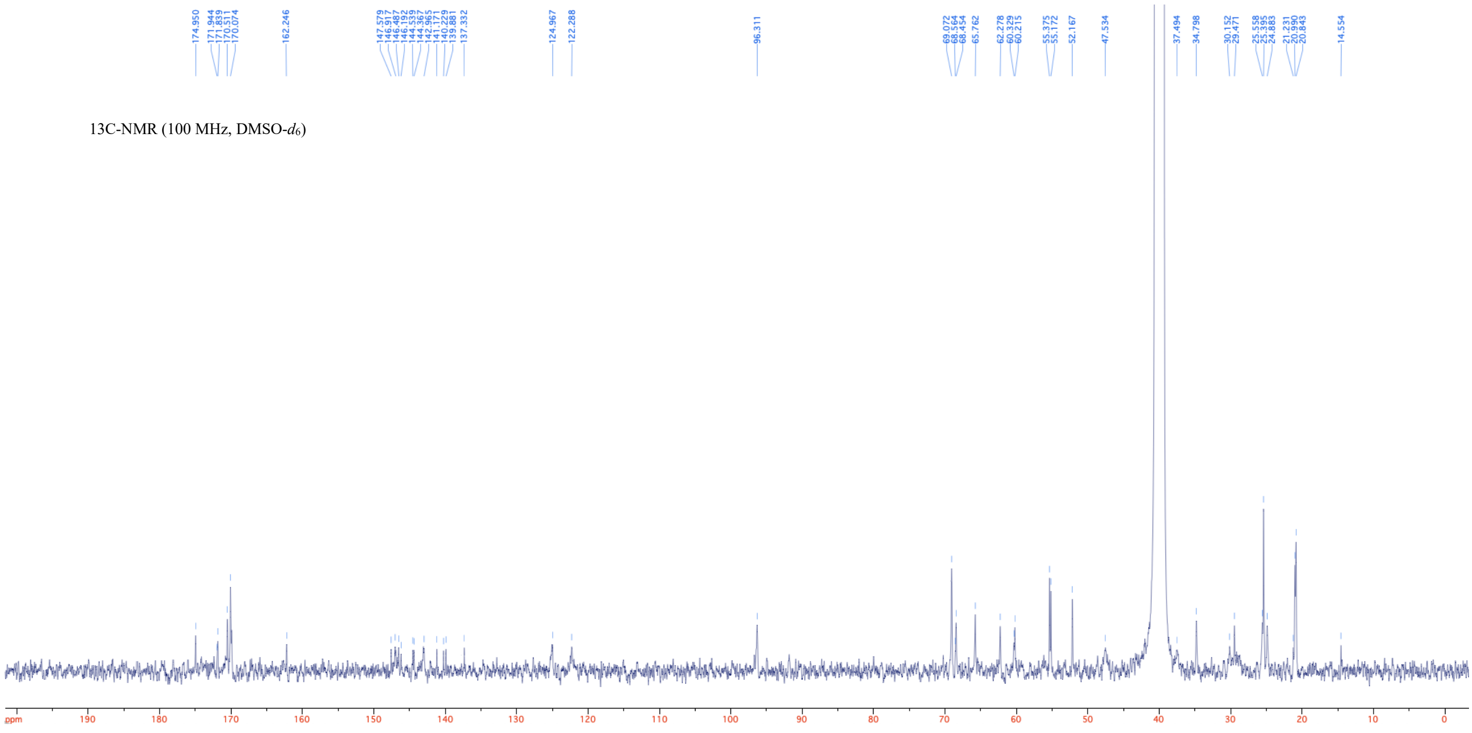

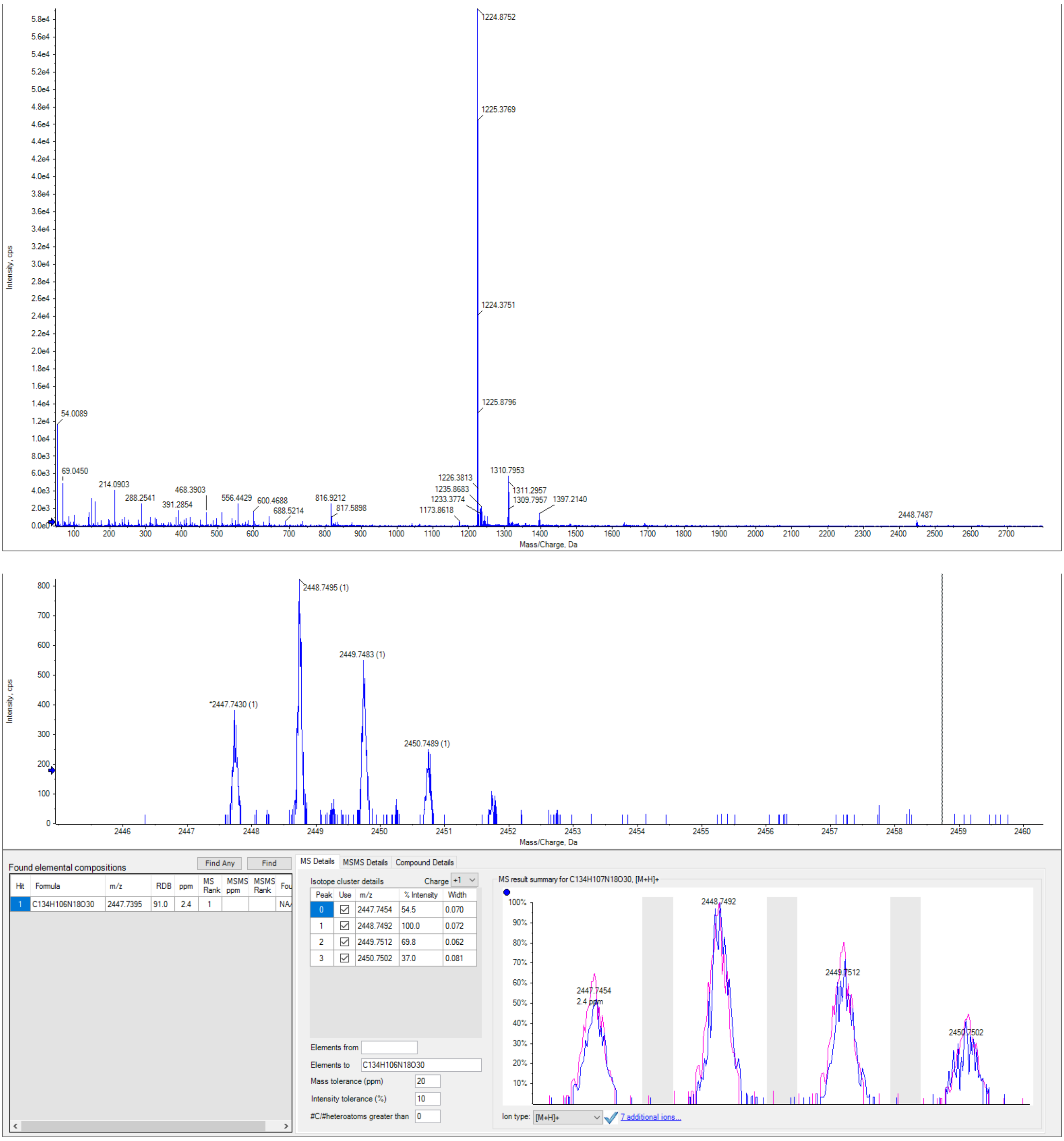
1224.8752
1225.3769
1224.3751
1225.8796
54.0089
69.0450
214.0903
288.2541
391.2854
468.3903
556.4429
600.4688
688.5214
816.9212
817.5898
1226.3813
1235.8683
1233.3774
1173.8618
1310.7953
1311.2957
1309.7957
1397.2140
2448.7487
Intensity, cps
Mass/Charge, Da
2448.7495 (1)
2449.7483 (1)
*2447.7430 (1)
2450.7489 (1)
Found elemental compositions
Find Any
Find
Hit
Formula
m/z
RDB
ppm
MS Rank
MSMS ppm
MSMS Rank
1
C134H106N18O30
2447.7395
91.0
2.4
1
NA
MS Details
MSMS Details
Compound Details
Isotope cluster details
Charge
+1
Peak
Use
m/z
% Intensity
Width
0
2447.7454
54.5
0.070
1
2448.7492
100.0
0.072
2
2449.7512
69.8
0.062
3
2450.7502
37.0
0.081
Elements from
Elements to
C134H106N18O30
Mass tolerance (ppm)
20
Intensity tolerance (%)
10
#C/#heteroatoms greater than
0
MS result summary for C134H107N18O30, [M+H]+
2447.7454
2.4 ppm
2448.7492
2449.7512
2450.7502
Ion type:
[M+H]+
7 additional ions...

UV-Vis spectrum

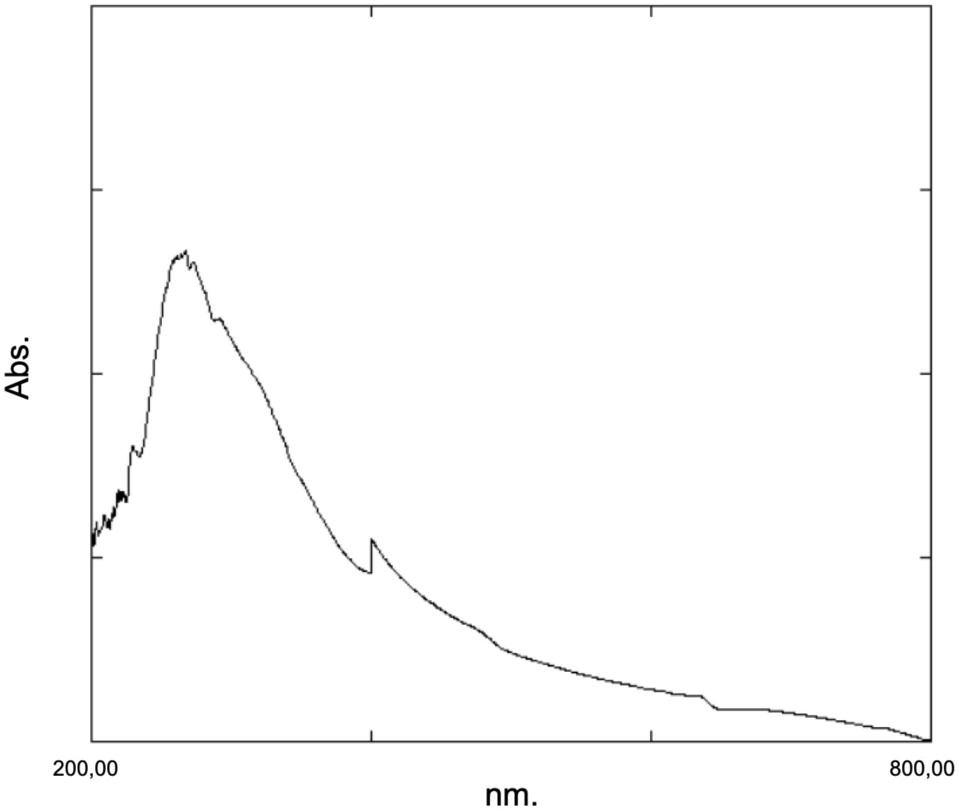

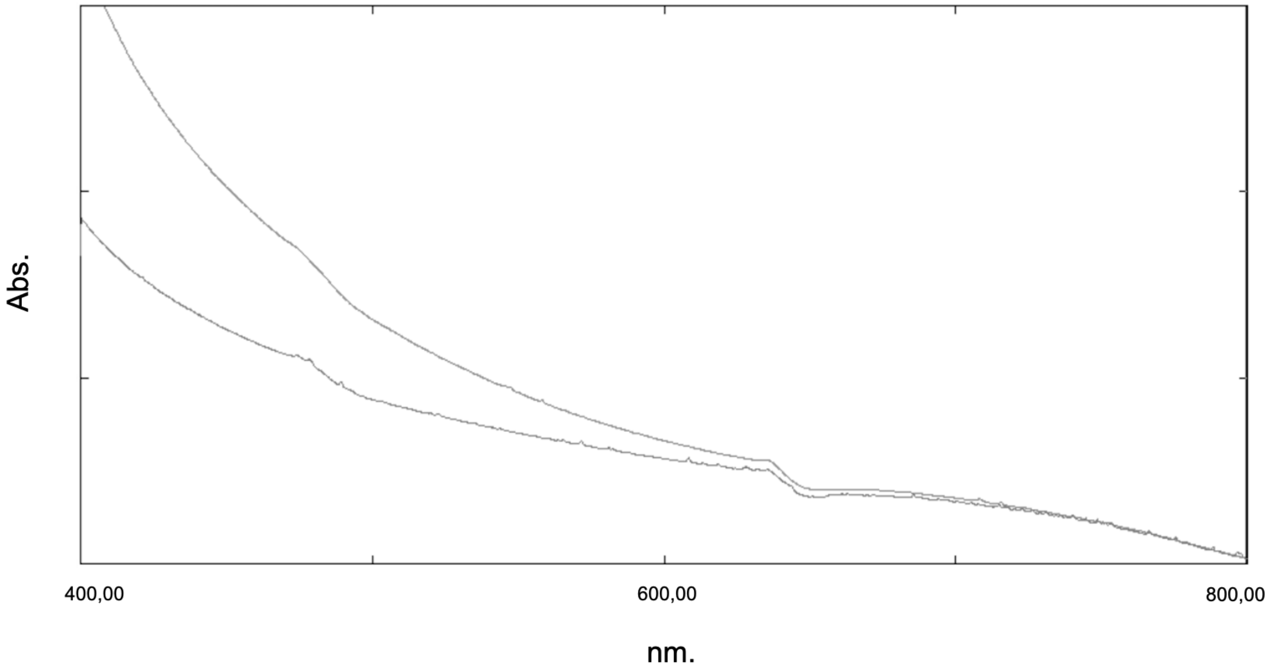

Supplement: Supplementary file 1 [file molecules-27-02776-s001.zip › molecules-1688607-supplementary.pdf]
